# ARHGEF9 regulates melanoma morphogenesis in environments with diverse geometry and elasticity by promoting filopodial-driven adhesion

**DOI:** 10.1016/j.isci.2022.104795

**Published:** 2022-08-08

**Authors:** Vicky Bousgouni, Oliver Inge, David Robertson, Ian Jones, Innes Clatworthy, Chris Bakal

**Affiliations:** 1 Division of Cancer Biology, Institute of Cancer Research, 237 Fulham Road, London SW3 6JB, UK; 2 Francis Crick Institute, 1 Midland Road, London NW1 1AT, UK; 3 Division of Breast Cancer Research, Institute of Cancer Research, 237 Fulham Road, London SW3 6JB, UK; 4 Core Research Laboratories, The Natural History Museum, Cromwell Road, London SW7 5BD, UK

**Keywords:** Cell biology, Biophysics, Cancer

## Abstract

Rho GTP Exchange Factors (RhoGEFs) and Rho GTPase Activating Proteins (RhoGAPs) are large families of molecules that regulate shape determination in all eukaryotes. In pathologies such as melanoma, RhoGEF and RhoGAP activity underpins the ability of cells to invade tissues of varying elasticity. To identify RhoGEFs and RhoGAPs that regulate melanoma cell shape on soft and/or stiff materials, we performed genetic screens, in tandem with single-cell quantitative morphological analysis. We show that ARHGEF9/Collybistin (Cb) is essential for cell shape determination on both soft and stiff materials, and in cells embedded in 3D soft hydrogel. ARHGEF9 is required for melanoma cells to invade 3D matrices. Depletion of ARHGEF9 results in loss of tension at focal adhesions decreased cell-wide contractility, and the inability to stabilize protrusions. Taken together we show that ARHGEF9 promotes the formation of actin-rich filopodia, which serves to establish and stabilize adhesions and determine melanoma cell shape.

## Highlights

• Screens on varying elasticity identify ARHGEF9 as a cell shape regulator
• ARHGEF9 depletion disrupts filopodia and focal adhesion morphogenesis
• Loss of ARHGEF9 results in a failure to generate contractile forces
• ARHGEF9 is essential for filopodia-driven protrusions in melanoma cells

## Graphical abstract

**Figure:**
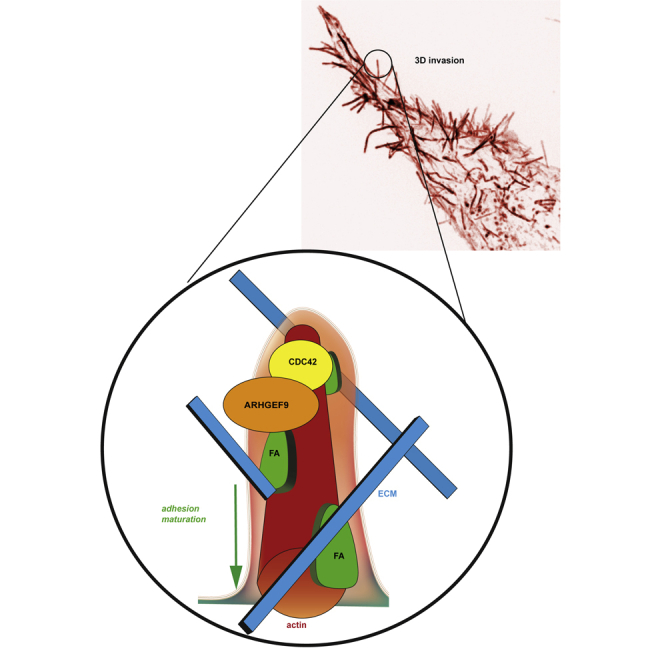

## Introduction

Despite advances in cancer research and therapies, malignant melanoma incidences are still on the rise. In 2020, 324,635 new cases of skin melanoma were reported worldwide (Sung et al., 2021). Malignant melanoma is highly metastatic and patients with advanced melanoma present a median survival of six to ten months, and a five-year survival of less than 5% (Erdei and Torres, 2010; Agarwala, 2009). Understanding the mechanisms controlling melanoma metastasis is thus essential for developing new therapies.

During metastasis, tumor cells undergo morphological changes in order to invade and migrate (Gardel et al., 2010; Gadea et al., 2008; Sanz-Moreno and Marshall, 2010; Yamada et al., 2019; Bakir et al., 2020). These morphological changes occur in response to mechanical and geometric cues from the extracellular matrix (ECM) that tumor cells encounter during metastasis (Rigato et al., 2015; Lämmermann and Sixt, 2009). In particular, tissue stiffness has a significant effect on cell shape (Charras and Sahai, 2014; Caswell and Zech, 2018). However, how metastatic cells alter their shape in response to mechanical and geometric cues is unknown.

In all eukaryotic cells, Rho GTPases regulate cell morphogenesis. Rho GTPases act as molecular switches that are activated when bound to GTP (Vega and Ridley, 2008; Ridley, 2015). Among Rho-family GTPases, CDC42 determines the shape of varying cell types by regulating actin polymerization and polarity (Heasman and Ridley, 2008). When recruited to the leading-edge membrane of migrating cells by cues such as phosphatidylinositol 4,5-bisphophate (PIP2), CDC42 regulates actin organization by different means. By activating Formin family proteins such as FMNL2, FMNL3, and mDia2, CDC42 can nucleate and elongate new actin filaments (Kage et al., 2017). CDC42 can also promote the branching of F-actin filaments by recruiting the Arp2/3 complex (Watson et al., 2017; Bonfim-Melo et al., 2018). Once nucleated by Arp2/3, filopodial actin filaments are bundled by crosslinking proteins such as Fascin (Jansen et al., 2011; Harker et al., 2019). The cell membrane is remodeled around filopodia by CDC42-mediated recruitment of IRSp53 (Mattila and Lappalainen, 2008; Vaggi et al., 2011). Moreover, IRSp53 promotes the clustering of the actin elongation factor VASP (Disanza et al., 2013). Bundling and membrane cross-linking promote the stability of filopodia structures (Schafer et al., 2011).

In some cell types, filopodia structures serve as templates that mark sites of adhesions to the substrate. For example, when cultured in 3D collagen hydrogels, endothelial cells make filopodia that guide angiogenesis *in vivo* (Gerhardt et al., 2003). In filopodia structures, the retrograde flow of F-actin promotes integrin cluster activation and engagement of the actin-adhesion “molecular clutch” which generates traction on the ECM at adhesion sites (Case and Waterman, 2015). The traction generated at filopodia via actin-adhesion complexes allows cells to move beyond the adhesion point (Noble et al., 2017). A similar process occurs during axonal pathfinding in 3D (Fischer et al., 2019). Importantly, owing to their high degree of interaction with the membrane in 3D, ECM-bound adhesions are likely to form across the surface of cylindrical filopodial structures. Filopodia-driven adhesions likely differ from those coupled with lamellipodial-driven focal adhesion (FA) structures such as observed in many migrating cells cultured on top of stiff 2D substrates (Jacquemet et al., 2015). In lamellipodia, FAs engage with branched actin resulting in a layered structure where actin is stacked on top of the FA forming a 2D interface (Case and Waterman, 2015). Owing to their shape, filopodia structures may be more capable of exploring shape space in 3D compared to largely 2D lamellipodia structures—allowing cells to find attachment points in multiple dimensions. But which signals regulate CDC42 itself to promote the formation of such structures is unclear. Potentially complexes that are responsive to matrix rigidity and geometry, effectively sensing the presence of a 3D substrate, could recruit and activate CDC42 to actin, cell membrane, and adhesions.

RhoGEFs promote the exchange of GDP for GTP thus activating Rho GTPases; while RhoGAPs catalyze the hydrolysis of GTP to GDP (Etienne-Manneville, 2004; Sadok and Marshall, 2014; Bos et al., 2007; Cherfils and Zeghouf, 2013). In humans, 82 RhoGEFS and 67 RhoGAPs regulate 20 Rho GTPases and their downstream effectors (Rossman et al., 2005). The Dbl RhoGEFs are multi-domain proteins that share a Dbl homology domain (DH) and an adjacent C-terminal Pleckstrin homology domain (PH). The exchange of GDP to GTP is catalyzed by the DH domain while the PH domain localizes the GEFs to the plasma membrane (Bos et al., 2007; Cook et al., 2014). Owing to their number and diversity, RhoGEFs and RhoGAPs serve as excellent candidates for genes that can be differentially activated in response to environment geometry and varying levels of matrix rigidity. In turn, RhoGEFs and RhoGAPs can dictate the spatiotemporal dynamics of Rho GTPase activation. Indeed, RhoGAPs such as CdGAP have been implicated in coupling matrix rigidity to the spatiotemporal dynamics of CDC42-mediated actin reorganization (Wormer et al., 2012).

Phenotypic screening approaches in combination with computational methods have been used to profile single-cell phenotypes and uncover the roles of RhoGEFs and RhoGAPs in the morphogenesis of cells on stiff 2D matrices (Bakal et al., 2007). But which RhoGEFs and RhoGAPs regulate shape on softer, more physiologically relevant, substrates remain unclear. We hypothesize that there are RhoGEFs and RhoGAPs which act to regulate shape on both soft and stiff surfaces, as well as “stiffness” specific RhoGEFs and RhoGAPs.

In order to investigate how RhoGEFs and RhoGAPs act to regulate shape in response to matrix rigidity, we performed RNAi screens of all RhoGEFs and RhoGAPs on WM266-4 melanoma cells cultured on soft (approx. 200 Pa) vs stiff (1 GPA) 2D materials (Weaver, 2017; van Helvert et al., 2018; Barnes et al., 2017). We show that WM266-4 cells cultured on top of 2D and 3D materials or embedded in 3D hydrogels form extensive filopodia protrusions. On stiff 2D materials, these filopodia mark sites of FA formation. Thus, WM266-4 cells bear morphological similarities to endothelial tip cells and neuronal sub-types. We found nine genes that regulate shape on soft materials, whereas five genes regulate shape on stiff. Only two genes, ITSN2 and ARHGEF9, both of which are CDC42 GEFs (Mitin et al., 2007; Rodriguez-Fraticelli et al., 2010), regulate melanoma shape on both soft and stiff materials.

We selected the CDC42 GEF (Harvey et al., 2004; Tyagarajan et al., 2011), ARHGEF9/Collybistin (Cb), for further exploration and to show how it regulates cell shape mechanistically. ARHGEF9 is essential for filopodia formation, FAs assembly, and contractility in cells cultured on 2D materials, and for protrusion in melanoma cells embedded in 3D matrices. In the absence of ARHGEF9-mediated filopodia formation, FA morphogenesis is disrupted, resulting in loss of traction at FAs. On 2D surfaces, this results in large flat cells, but in 3D matrices depletion of ARHGEF9 results in rounded forms. We show that ARHGEF9’s exchange activity on CDC42 is required for its role in cell shape determination and adhesion morphogenesis. We propose that melanoma cells utilize ARHGEF9 to promote filopodial-driven adhesion during morphogenesis in environments with varying rigidity and geometry. ARHGEF9 may thus confer melanoma cells with the ability to invade diverse tissue during metastasis in a similar manner to how endothelial or neuronal cells migrate through the body during development.

## Results

### WM266-4 cells exhibit robust filopodial protrusions on top of stiff and soft materials

**Figure 1:**
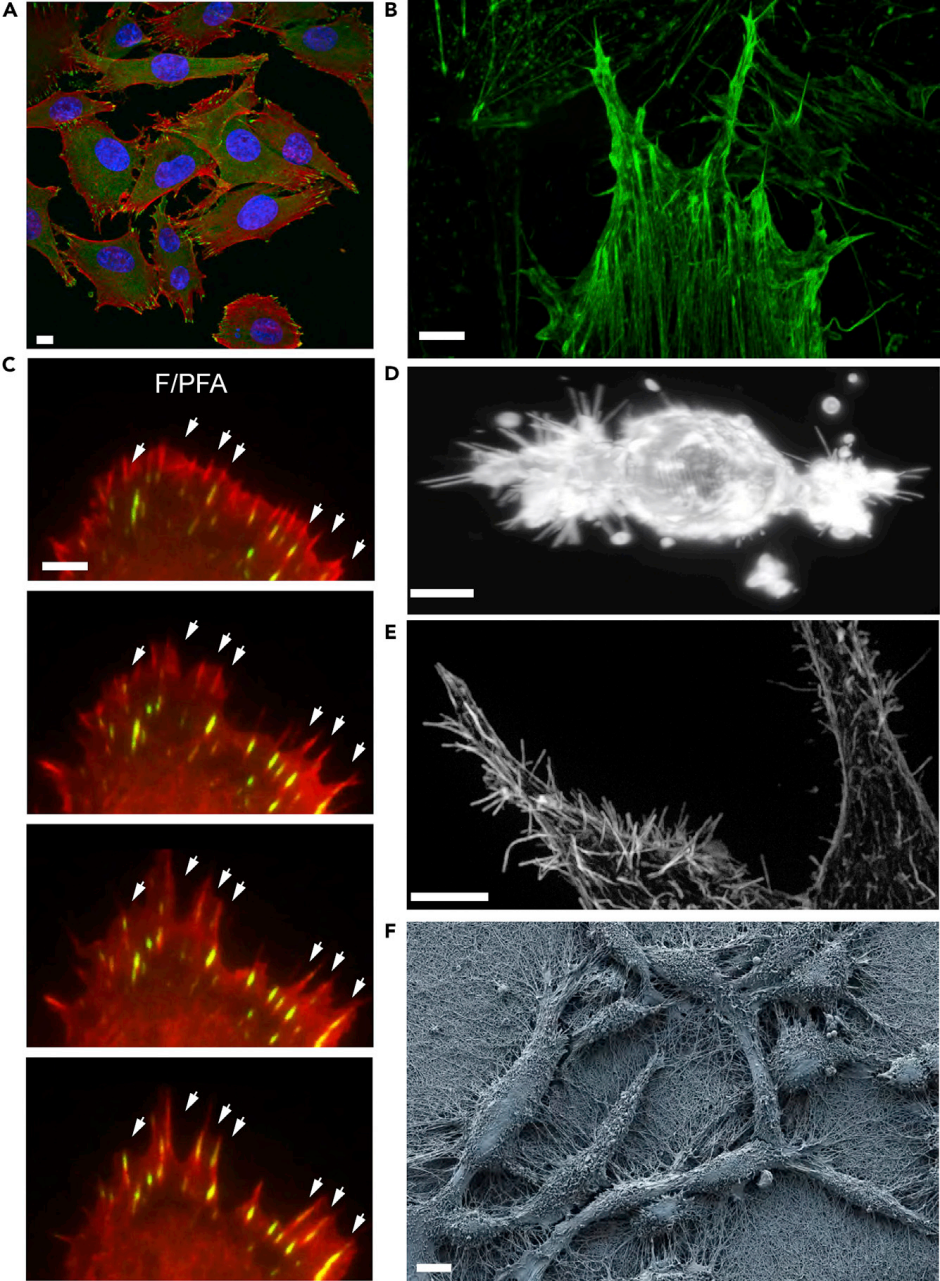
WM266-4 cells form filopodia-rich protrusions on both soft and stiff materials (A) Representative confocal microscopy image of WM266-4 cells cultured on top of plastic, stained for F-actin (red), Paxillin (green), and Hoescht (blue). Scale bars, 10 μm. (B) Representative SoRa microscopy image of WM266-4 filopodia structures. Stained F-actin (green), scale bars 10 μm. (C) Representative TIRF microscopy images of a WM266-4 cell overexpressing Talin-mApple (green) and LifeAct-Mars (red). Scale bars, 10 μm. (D) Representative lattice light sheet microscopy image of a WM266-4 cell embedded in 3 mgs/mL collagen. Scale bars, 5 μm. (E) Representative SoRa microscopy image of WM266-4 cells showing filopodia structures. Stained for F- actin (red). Scale bars, 10 μm. (F) Representative scanning electron microscopy image of WM266-4 cells cultured on top of soft collagen, forming elongated protrusions anchored by collagen fibers. Scale bar, 10 μm.

Our goal was to identify mechanisms that regulate melanoma cell shape determination in response to matrix rigidity. As a model, we used WM266-4 cells. These are BRAF-driven melanoma that has been previously characterized to stochastically switch between round/ameboid/contractile shapes and spindle/mesenchymal/protrusive forms when embedded (Sahai and Marshall, 2003), or cultured on the surfaces of collagen hydrogels (Cooper et al., 2015; Yin et al., 2013). After long-term (24h) culturing on 2D stiff material, WM266-4 assumed a spread morphology (Figure 1A). WM266-4 cells rarely displayed lamellipodia leading edge and/or contractile rear, but rather appeared to be often unpolarized or bipolar (Figure 1A). On 2D stiff material, WM266-4 cells formed filopodia-rich protrusions which anchored stress fibers that often ran parallel to the long axis of the cells (Figures 1A and 1B). At these protrusions, Paxillin positive FAs formed at the base of filopodia which anchored stress fibers (Figure 1A).

To study actin and FA dynamics, we first transfected WM266-4 cells with Talin-mApple, and LifeAct-Mars (Riedl et al., 2008) and plated them on collagen-coated coverslips. 3h after plating, WM266-4 cells rapidly spread and formed extensive filopodia protrusions (Figure 1C). These protrusions marked the site of FA formation, as Talin accumulated to actin-dense regions in the filopodia minutes after their formation. Over time, small lamella often formed which bridged individual lamellipodia-like webbing between fingers (Figure 1C).

When embedded in 3D soft collagen hydrogels, WM266-4 cells resembled “wrapped candy” with a rounded cell body that bore two bipolar protrusions (Figure 1D). As in 2D, WM266-4 protrusions were very rich in filopodia and small lamella structures that bridged individual filopodial structures. When cultured on top of soft 2D hydrogels, WM266-4 formed extensive filopodia protrusions bridged by small lamella (Figure 1E). By scanning electron microscopy (SEM), filopodia protrusions appeared to be anchored by collagen fibers (Figure 1F). Taken together these data suggest that WM266-4 cells form protrusions in both 2D and 3D, and across matrix rigidities. In cells on 2D stiff material, filopodia are anchored to substrates by FAs which also engage actin stress fibers. The filopodia-rich protrusions without an obvious leading edge of WM266-4 are more like neuronal cell types than fibroblasts or keratocytes, (Yin et al., 2013; Keren et al., 2008).

### Identification of Rho GTP exchange factors and Rho GTPase activating proteins that regulate cell shape determination on soft and stiff materials

**Figure 2:**
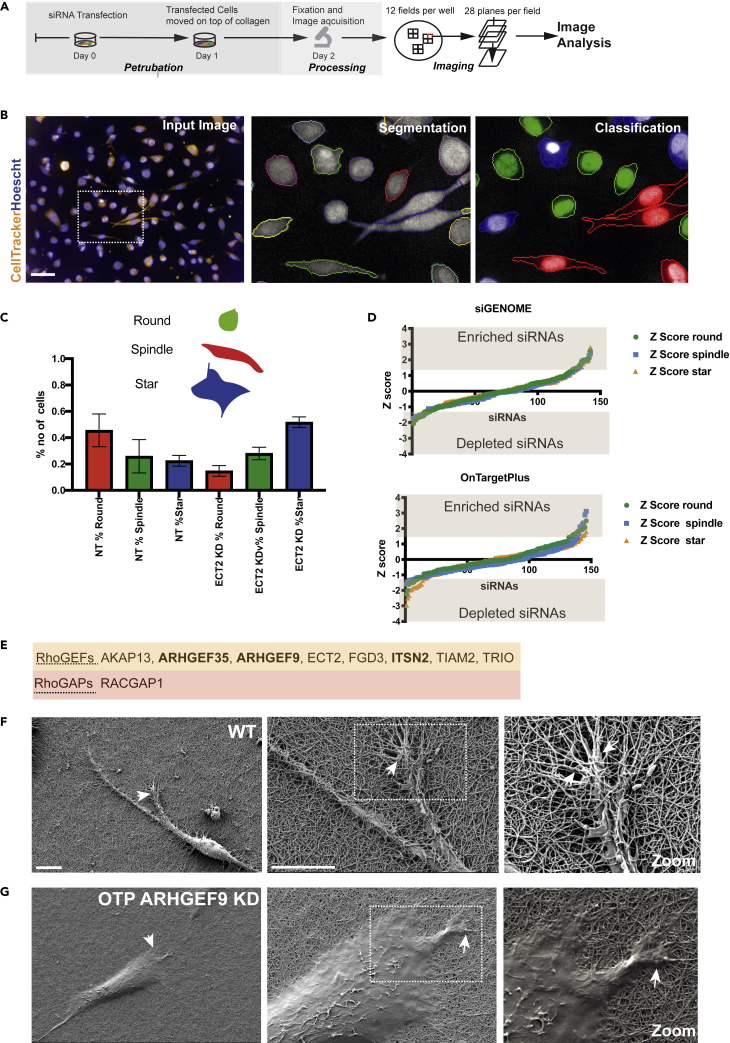
Automated siRNA phenotypic screens to identify RhoGEFs and RhoGAPs that regulate cell shape on top of soft hydrogel (A) Workflow: WM266-4 melanoma cells were labeled with CellTracker Orange and transfected with siRNAs in duplicate. Cells were transferred on top of 2 mgs/mL rat tail collagen and 24h later were fixed and stained with Hoescht (blue). Twelve fields across twenty-eight planes per well were imaged with automated confocal microscopy. (B) Image Analysis: The maximum projection was used for automated cell segmentation, texture and morphological features were calculated. Cells were classified into three shapes. Scale bars, 50 μm. (C) Cell shape: A linear classifier was trained to separate cells into three different shapes: round, spindle, and star. Cells transfected with non-targeting siRNA were heterogeneous, in contrast, ECT2 depletion enriched “star” phenotype. (D) Graphs depicting Z-scores of percentages of each shape in the population following knockdown of RhoGEFs and RhoGAPs. (E) Nine siRNAs were hits for the enrichment of one shape. (F) Representative scanning electron microscopy images of WT and ARHGEF9 depleted cells on top of rat tail type I collagen. Scale bars 10 and 2 μm.

We developed a phenotypic screen where we depleted the majority of human RhoGEFs and RhoGAPs using siRNA in WM266-4 melanoma cells cultured on top of 1.8 mgs/mL rat tail collagen type I (Col-I) hydrogel. The elasticity of this gel is approximately 100-200 Pa and is considered a “soft” material, i.e. has a similar stiffness to brain tissue (Butcher et al., 2009; Barnes et al., 2017). We used two different libraries: the siGenome library (Dharmacon) of 142 siRNAs targeting 77 RhoGEFs, 54 RhoGAPs, and 11 DOCKs; and the OnTargetPlus (OTP) library (Dharmacon) of 147 siRNAs targeting 77 RhoGEFs, 59 RhoGAPs and 11 DOCKs (Tables S1: OnTargetPlus (OTP) library, and S2: siGenome library. Both tables related to STAR methods). The screens were performed in quadruplicate. Non-targeting (NT) controls were included in all plates. Cytoplasmic and nucleus regions were automatically segmented, and we quantified 28 morphological and texture features from maximum projections of z-planes (Table S3) (Figure 2A).

To describe the cell shapes of different populations, we generated “Population-levelQuantitative Morphological Signatures” (pQMS) (Bakal et al., 2007). Each pQMS describes the proportion of cells that can be described as having similar exemplar shapes. Our exemplar shapes in this screen were initially determined based on qualitative observations; “round”—where the cells have no protrusions; “spindle”—where cells have a typical elongated body with two protrusions, and a “star” like morphology with three or more protrusions (Figure 2B). Linear classifiers were then generated which can distinguish each shape. In this context, a pQMS is a feature vector comprised of the percentage of cells described as either “round,” “spindle,” or “star.” We transfected two 96 well plates with non-targeting siRNAs and observed that control populations were highly heterogeneous in terms of shape: 45% of the population were classified as round, 26% spindle, and 23% star shapes (Figure 2C). To confirm transfection efficiency, we transfected cells with ECT2 RNAi which inhibits cytokinesis, resulting in changes in cell morphology (Pascual-Vargas et al., 2017). Indeed, ECT2 depletion significantly altered the proportion of round, spindle, and star cells in the population, with 51% of the cells were classified as star shaped. Of the siRNAs, 76% of siRNAs and 82% from the OTP library, respectively, did not enhance or suppress a specific shape. Thus, most siRNAs in the libraries do not affect the shape of WM266-4 melanoma cells.

Candidate siRNA of interest were selected when at least two replicates had a *Z* score above a threshold of 1.5 or below −1.5 (Figure 2D). We identified 34 siRNAs from the siGenome pool library and 25 siRNAs from the OTP pool library which affected the proportion of at least one exemplar sub-population (Tables S4, S5). Nine siRNAs were hits across both libraries: AKAP13, ARHGEF35, ARHGEF9, ECT2, FGD3, ITSN2, RACGAP1, TIAM2, and TRIO (Figure 2E) (Figure S1A).

Depletion of ARHGEF9 was particularly effective at altering cell shape in melanoma cells on soft material. We noticed 48.7 and 46.5% increases in the proportion of star and spindle-shaped cells following knockdown by siGENOME and OTP reagents, respectively. We validated that siRNAs that targeted ARHGEF9 effectively depleted ARHGEF9 RNA, and protein (Figures S2A and S2B). To validate our findings from the screen we performed scanning electron microscopy (SEM) of wild-type (WT) and ARHGEF9 depleted cells cultured on top of collagen. We depleted ARHGEF9 with siRNA (OTP Library) and after 48h, samples were fixed and transferred on stubs for SEM. WT cells on collagen exhibited elongated shapes with extensive filopodia, and lamella protrusions (Figure 2F). The cell body of WM266-4 cells was highly contractile (Figure 2F). Protrusions in these cells interacted extensively with collagen fibers. For example, WM266-4 cells appeared to “pull” on collagen fibers (Figure 2F). Moreover, collagen fibers were wrapped around control cells (Figure 2F). In contrast, ARHGEF9 depleted cells appeared to have a shorter flat shape and did not extend long protrusions with extensive filopodia and lamellipodia (Figure 2G). In addition, ARHGEF9 depleted cells did not appear to interact with the collagen fibers rather ARHGEF9 cells appear to “float” above the collagen matrix (Figure 2G).

**Figure 3:**
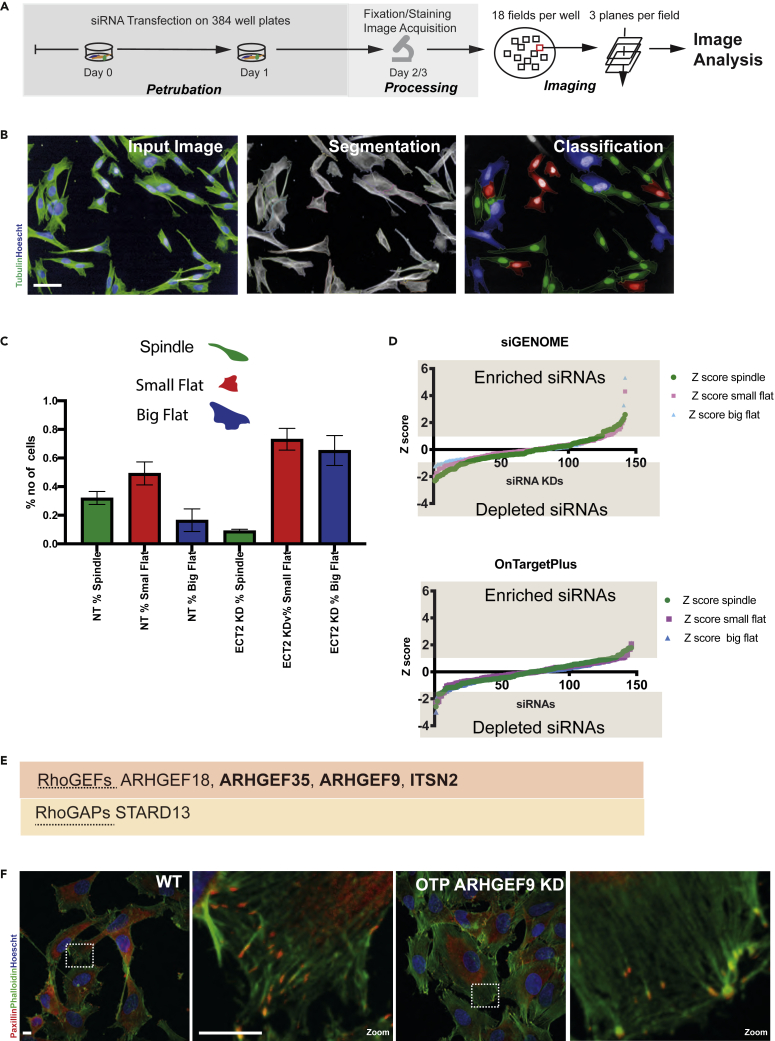
siRNA screens to identify RhoGEFs and RhoGAPs that regulate cell shape on top of the stiff substrate (A) Workflow: WM266-4 melanoma cells were transfected with 142 siRNAs from the siGENOME and 147 siRNAs from the OnTargetPlus libraries. All samples were duplicated in 384 well plates and replicate plates were included. Following 48h incubation, cells were fixed and stained with α-tubulin (red) and Hoescht (blue). Plates were imaged with automated confocal microscopy. (B) Image Analysis: Maximum projection was used for automated cell segmentation, texture and morphological features were calculated. Cells were classified into three shapes. Scale bars, 50 μm. (C) Cell shape: A linear classifier was trained to separate cells into three different shapes: spindle, small flat, and big flat. Cells transfected with non-targeting siRNA were heterogeneous, in contrast, ECT2 depletion enriched “small/big flat” phenotype. (D) Graphs depicting Z-scores of percentages of each shape in the population following knockdown of RhoGEFs and RhoGAPs. (E) Five siRNAs were hits for the enrichment of one shape. (F) Representative images of WT and ARHGEF9 depleted cells stained for F-actin (green), Paxillin (red), and Hoescht (blue). Scale bars, 10 μm.

To determine if RhoGEFs and RhoGAPs that regulate morphogenesis on soft matrices also contribute to cell shape determination on stiff substrates, we repeated the screens using WM266-4 cells cultured on 2D plastic (Figure 3A). Cells were stained for α-tubulin and DNA for automated high-content imaging. Morphological and texture features (Table S6) were used to train three linear classifiers for three exemplar 2D shapes: “spindle,” “small flat,” and “big flat” phenotypes (Figure 3B). pQMSs were generated that described the proportion of sub-populations with each of these three shapes. Note that these shapes were quantitatively different than those identified on soft materials. As on soft materials, control populations were a heterogeneous mixture of shapes. Specifically, WT cells were comprised of 32% spindle, 49% small flat, and 16% big flat phenotypes (Figure 3C). From, the siGenome library we identified 30 siRNAs and 28 siRNAs from the OTP library, enriching at least one shape (Tables S7, S8), (Figure 3D). The percentage of siRNAs with positive and negative scores for a shape was 36% in siGenome whilst in the OTP screen, it was only 20%. From the 2D screens, five siRNAs were hits across both libraries: ARHGEF18, ARHGEF35, ARHGEF9, ITSN2, and STARD13 (Figures 3E and S1B). ARHGEF9 depleted cells enriched the big flat phenotype for both libraries. When comparing all candidate genes across all screens in both soft and stiff matrices, only two siRNAs, ARHGEF9 and ITSN2 led to consistent changes in cell morphology.

**Figure 4:**
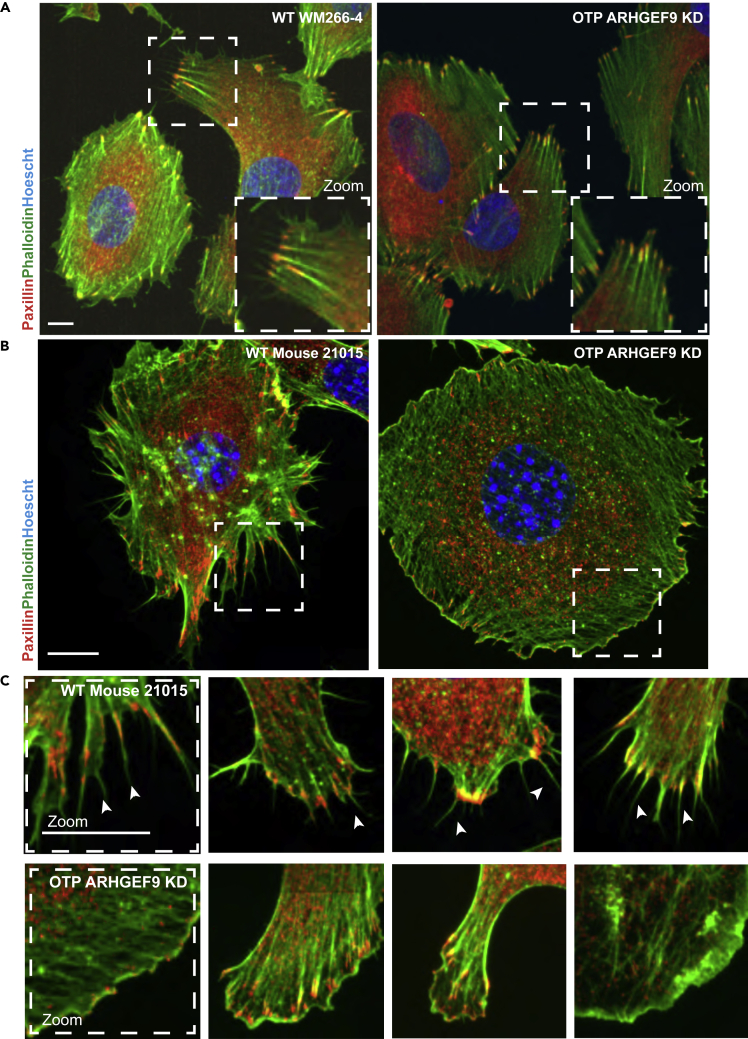
Human WM266-4 and mouse 21015 melanoma cells form filopodia-rich protrusions on top of stiff materials (A) Representative confocal microscopy images of human WM266-4 melanoma and ARHGEF9 depleted cells cultured on top of stiff material, stained for F-actin (red), Paxillin (green), and Hoescht (blue). Scale bars, 10 μm. (B) Representative confocal microscopy images of WT 21015 mouse melanoma and ARHGEF9 depleted cells cultured on top of stiff material, stained for F-actin (red), Paxillin (green), and Hoescht (blue). Scale bars, 10 μm. (C) Zoomed images showing filopodia structures of WT21015 mouse melanoma. ARHGEF9 depleted cells lack filopodia. Scale bars, 10 μm.

As a CDC42 GEF (Harvey et al., 2004), we hypothesized that ARHGEF9 mediated the activation of CDC42 promotes the formation of filopodia (Ridley, 2011). Because filopodia mark the sites of future adhesion formation in WM266-4 melanoma cells (Figure 1C), loss of ARHGEF9 filopodia could alter adhesion morphogenesis, resulting in morphogenesis defects on stiff 2D plastic and in 3D hydrogels. To investigate this hypothesis, we imaged F-actin and FAs in WT and ARHGEF9 depleted cells cultured on 2D. FAs were labeled using anti-Paxillin antibody (Methods). Proliferating WM266-4 cells on plastic made extensive small FAs at the base of filopodial protrusions that emanated from the front of the cells. These FAs also anchored thick F-actin stress fibers (Figure 4A). In contrast, ARHGEF9 cells were largely devoid of filopodia, though still made stress fiber-like structures that were anchored at larger FAs (Figure 4A).

To determine if ARHGEF9 had a function in filopodial morphogenesis in other cell types, we inhibited ARHGEF9 in mouse cutaneous melanoma 21015 cells. These cells harbor an activating BRAF mutation V600E (Dhomen et al., 2009). Like WM266-4 cells, 2105 cells are highly protrusive, neuronal-like cells, that form extensive thin filopodia structures when cultured on 2D plastic. These filopodia often emanated from peripheral adhesions (Figure 4B). Depletion of ARHGEF9 resulted in a striking change in morphology, F-actin organization, and adhesion morphogenesis (Figures 4B and 4C). ARHGEF9 depleted 21015 cells were large, flat, poorly contractile. Moreover, these cells often exhibited no filopodia, and had poor adhesion formation. Thus, we propose that ARHGEF9 has a role in filopodia and adhesion morphogenesis in different cell types.

### ARHGEF9 promotes the extension of protrusions in response to changes in geometrical cues

**Figure 5:**
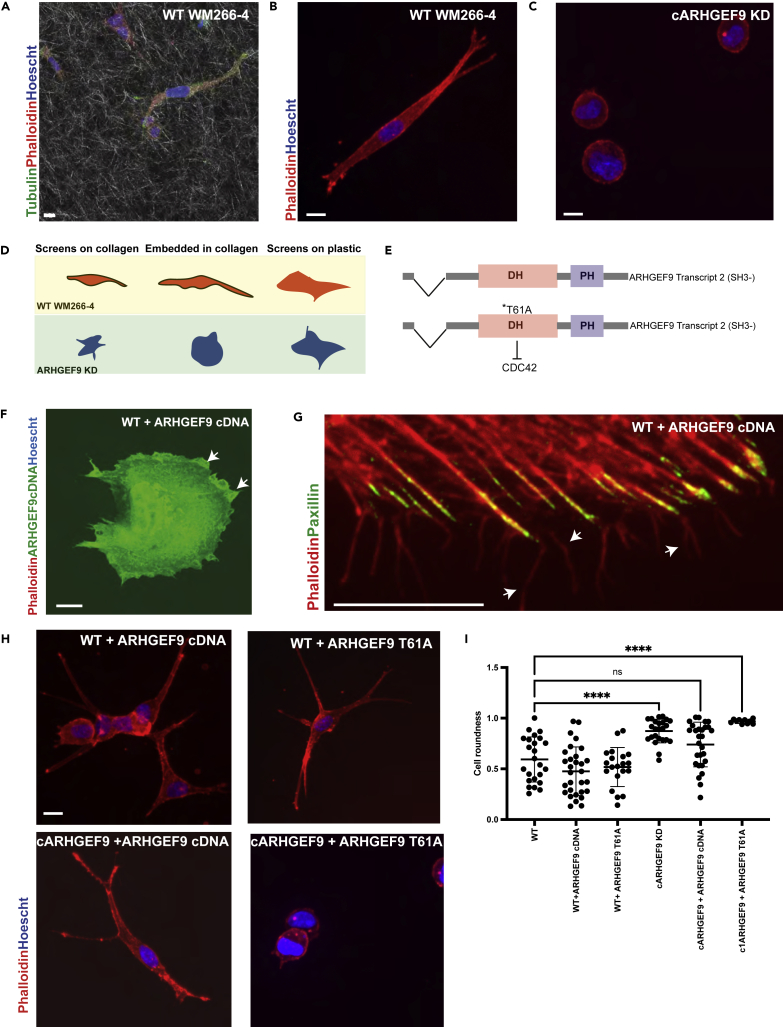
Overexpression of ARHGEF9 cDNA on WM266-4 rescues cell protrusions (A–C) Representative images of WM266-4 cells embedded in collagen (visible collagen fibers) and WT, ARHGEF9 depleted cells (custom siRNA ARHGEF9, cARHGEF9), Scale bars 10 μm. (D) Schematic representation of WT and ARHGEF9 depleted cells on different stiffnesses. (E) Schematic representation of ARHGEF9 constructs. (F)Representative image of WT overexpressing ARHGEF9 cDNA. Scale bar 10 μm. (G) Representative SoRa microscopy image of WT overexpressing ARHGEF9 cDNA showing filopodia structures at the edge of the cell. Scale bar 10 μm. (H) Representative images of WM266-4 embedded in collagen showing WT overexpressing ARHGEF9 cDNA, WT overexpressing ARHGEF9 T61A, ARHGEF9 depleted cells, ARHGEF9 overexpressing ARHGEF9 cDNA and ARHGEF9 depleted overexpressing ARHGEF9 T61A. Scale bars 10 μm. (I) Graph depicting cell roundness of single cells in all conditions. Comparison between groups was performed by One-way ANOVA with prism9 software (WT vs ARHGEF9 KD cells ∗∗∗∗p < 0.0001 and WT vs ARHGEF9 T61A ∗∗∗∗p < 0.0001).

We next tested the role of ARHGEF9 in cells embedded in the collagen hydrogel matrix. Although the predicted stiffness cells feel when embedded is likely to be similar to that when cultured on the surface, the ECM geometry is radically different (Figure 4A) (Kadler et al., 2007; Shoulders and Raines, 2009). In 3D, WT WM266-4 cells typically exist in spindle shapes and make large protrusions (Figure 5A). Thin filopodia emerge from the protrusion body (Figure 5B). In contrast, ARHGEF9 depleted cells (by custom-designed siRNA, cARHGEF9) were largely round and poorly protrusive (Figure 5C). Thus, the shape of ARHGEF9 depleted cells varies depending on matrix stiffness and ECM geometry (Figure 5D).

To validate the role of ARHGEF9 in 3D cell shape determination we sought to rescue the phenotype of ARHGEF9 depleted cells. We used a constitutively active ARHGEF9 cDNA (“transcript 2”; lacking the SH3 domain) that is capable of GTP exchange activity on CDC42 (Figure 5E) (Harvey et al., 2004; Soykan et al., 2014; Reddy-Alla et al., 2010). Overexpressed constitutively active ARHGEF9-GFP localized primarily to the cytoplasm was enriched at the cell membrane (Figure 5F) and induced extensive filopodia formation (Figure 5G).

We also expressed a mutant ARHGEF9 cDNA transcript which harbors a T61A mutation. Threonine 61 is critical for the interaction of ARHGEF9 with CDC42 and thus its GEF function (Figure 5F) (Reddy-Alla et al., 2010). Expression of constitutively active ARHGEF9 cDNA led to multiple large protrusions. The tips of these protrusions often bore tubulin-rich puncta that resembled synaptic boutons (Ruiz-Canada et al., 2004). Similar to the expression of constitutively active ARHGEF9 cDNA, the expression of mutant ARHGEF9 T61A promoted the formation of protrusions (Figure 5H). These protrusions, however, were significantly thinner and did not have tubulin-rich puncta at the tips. Thus, ARHGEF9 promotes protrusion formation in 3D in a CDC42-dependent fashion. But there is also CDC42 independent roles for ARHGEF9 in 3D cell shape determination.

Cells depleted of ARHGEF9 by a custom-designed siRNA (cARHGEF9) expressing WT ARHGEF9 cDNA, were elongated and largely bipolar, showing a significantly more protrusive phenotype than cARHGEF9 depleted cells alone. Overexpression of ARHGEF9 T61A in ARHGEF9 depleted cells led to no protrusive activity and cells remained rounded (Figure 5H). To quantify the differences in shape between conditions, we measured the cell roundness of single cells across all conditions. Cells overexpressing ARHGEF9-GFP showed no significant difference from WT cells. In contrast, cARHGEF9 depleted cells and cARHGEF9 overexpressing ARHGEF9 T61A were significantly rounder than WT cells (∗∗∗∗p < 0.0001) (Figure 5I). Thus, ARHGEF9 acts as a GEF to regulate the morphogenesis of cells cultured on both soft and stiff 2D materials, as well as in 3D environments. In particular, ARHGEF9 is essential for the formation of extensive protrusions that interact with collagen fibers. Notably, as ARHGEF9 depleted cells on 2D surfaces are “flat” and poorly contractile; we postulate that ARHGEF9’s role in promoting protrusions in 3D must in part be owing to the upregulation of attachment and/or traction that drives contractile forces.

### ARHGEF9 regulates focal adhesion morphogenesis

We hypothesized that ARHGEF9-CDC42 could promote filopodia formation which could then serve as platforms for the assembly of FAs, a behavior we observed during cell spreading (Figure 1C). Engagement of filopodia actin at FAs could then generate traction. If so, we predicted the depletion of ARHGEF9 in WM266-4 cells would result not only in loss of filopodia, but defects in FA morphogenesis.

**Figure 6:**
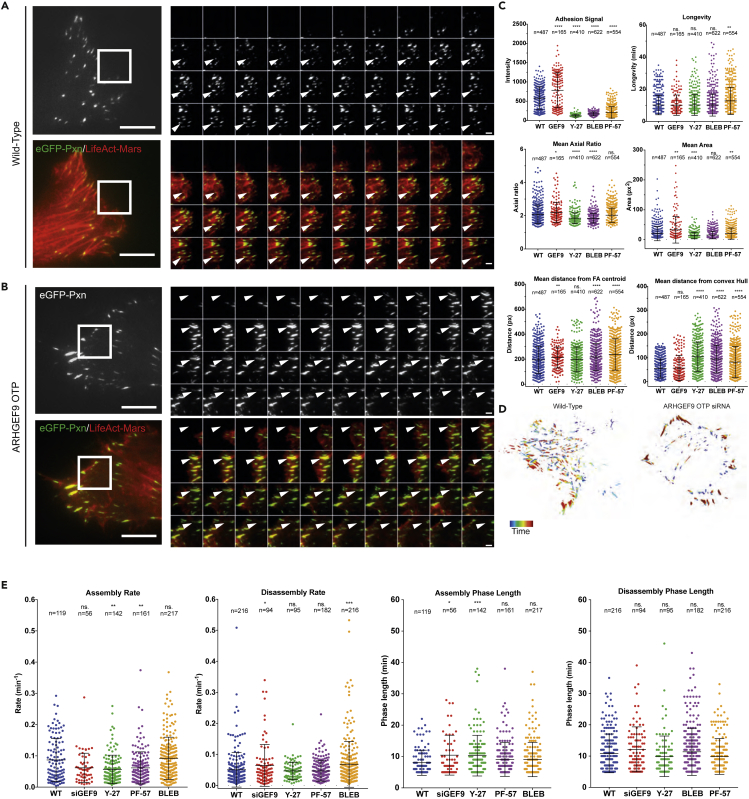
ARHGEF9 regulates adhesion dynamics in WM266-4 cells (A and B) Representative total internal reflection fluorescence microscopy images of a WM266-4 cell and ARHGEF9 depleted cell expressing eGFP-Paxillin and LifeAct-Mars for actin. (C) Single lineage features output from the focal adhesion analysis server, wild-type (n = 487), ARHGEF9 OTP siRNA (n = 165), Y-27632 (n = 410), Blebbistatin (n = 622) and PF573288 (n = 554). Paxillin dynamics visualizations showing adhesion dynamics over time in wild-type and ARHGEF9. (D) Paxillin dynamics visualizations showing adhesion dynamics over time in WT and OTP ARHGEF9 KD cells. (E) Adhesion kinetics data measuring assembly and disassembly rates as well as the length of each phase, calculated using the FAAS. Assembling adhesions that were detected were as follows, Wild-type (n = 119), ARHGEF9 OTP siRNA (n = 56), Y-27632 (n = 142), Blebbistatin (n = 161) and PF-573288 (n = 217). Disassembling adhesions that were detected were as follows, Wild-type (n = 216), ARHGEF9 OTP siRNA (n = 94), Y-27632 (n = 95), Blebbistatin (n = 182) and PF-573288 (n = 216).

We sought to characterize adhesion and actin dynamics in WT WM266-4 (Figure 6A) and ARHGEF9 OTP siRNA treated cells (Figure 6B) as they spread on collagen coated in 2D using total internal reflection fluorescence microscopy (TIRF).To track adhesions, we transiently overexpressed an N-terminally tagged eGFP-Paxillin. F-actin was visualized through overexpression of LifeAct-Mars (Riedl et al., 2008). Cells were imaged between 1 and 8h after plating which enabled us to capture adhesion dynamics as cells first spread. Using the Focal Adhesion Analysis Server (FAAS), we segmented TIRF images and extracted features such as adhesion intensity, longevity, axial ratio, mean area, mean distance from the centroid, and mean distance from convex hull (Figure 6C) (Berginski and Gomez, 2013; Berginski et al., 2011). Additionally, kinetic parameters such as assembly/disassembly rates and assembly/disassembly phase lengths were calculated to identify specific changes in FAs turnover kinetics (Figure 6E).

To characterize FA dynamics in wild-type WM266-4 cells, and better understand the effects of ARHGEF9 depletion we also studied the effects of three small molecule inhibitors targeting different stages of FA formation and maturation: the ROCK inhibitor Y-27632, the myosin II Inhibitor Blebbistatin, and the focal adhesion kinase (FAK) inhibitor PF-573288. During spreading, wild-type WM266-4 cells have rapidly forming focal complexes, which quickly mature into larger FAs (Figure 6C). Inhibition of actomyosin contractility by Y-27632 and Blebbistatin, required for FA maturation, resulted in a decrease in Paxillin intensity at adhesions, axial ratio, and adhesion area (Figure 6C). ROCK and Myosin II inhibition also lead to slower assembly rates whilst ROCK inhibition additionally increased the mean length of FA assembly phases (Figure 6C). Thus, we conclude that in WM266-4 cells, ROCK-myosin-mediated maturation of FAs is a major determinant of FA morphogenesis.

Inhibition of FAK kinase activity also reduced FA intensity and area (Figure 6C). Additionally, PF-573288 treatment increased mean adhesion longevity (mean 1.25-fold increase, p < 0.005), consistent with the role of FAK in promoting adhesion turnover (Webb et al., 2004). However, the effects of PF-573288 on FAs in WM266-4 cells were surprisingly mild, for example, compared to those seen in epithelial cell lines (Sero and Bakal, 2017). Taken together, these results demonstrate that FAK is not a major regulator of adhesion dynamics in WM266-4 cells.

Compared to wild-type cells, ARHGEF9 knockdown resulted in an increase in mean FA area (mean 1.62-fold increase, p < 0.00005), mean axial ratio (mean 1.04-fold increase, p < 0.05) and mean Paxillin signal (mean 1.35-fold increase, p < 0.00005) (Figure 6C). FAs in ARHGEF9 knockdown were, on average, further away from the FA centroid (p < 0.005), however, not significantly further away from the convex hull compared to WT (Figure 6C). Thus, ARHGEF9 depletion leads to the formation of large, long, Paxillin-enriched cortical FAs (Figure 6D). ARHGEF9 depletion resulted in a shift in both FA assembly rate and phase length. Following ARHGEF9 depletions FAs were slower to assemble overall, and quick assembling adhesions were largely absent (Figure 6E).

**Figure 7:**
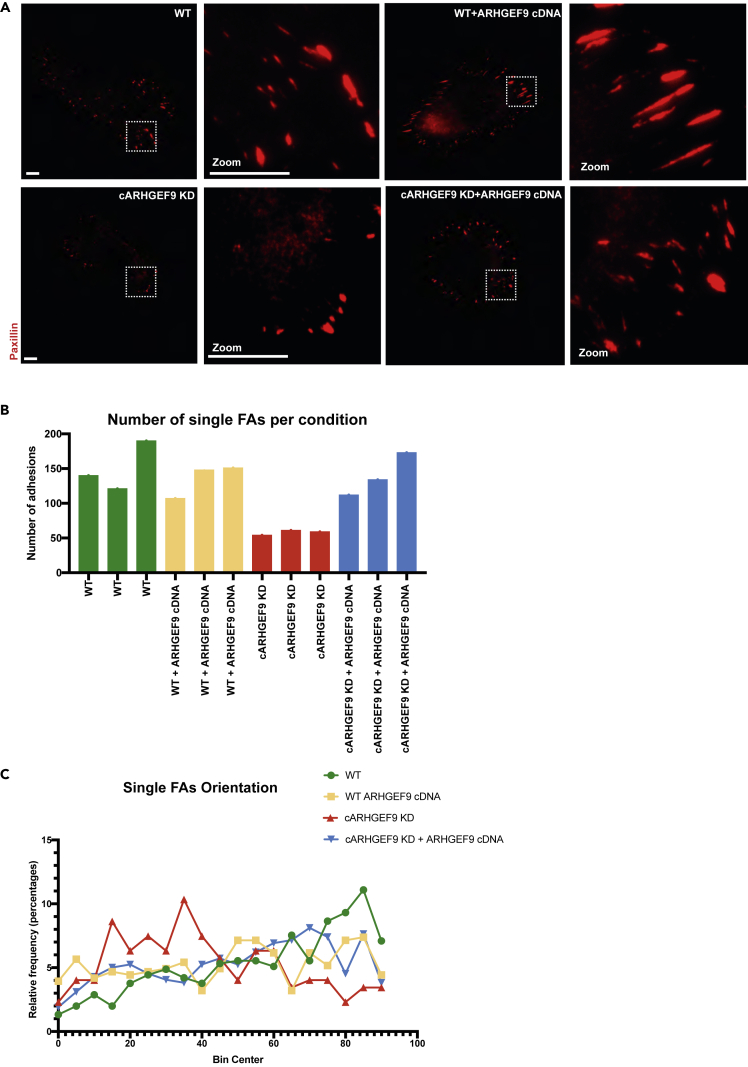
ARHGEF9 regulates FAs localization (A) Representative images of FAs in WT, WT overexpressing ARHGEF9 WT, ARHGEF9 depleted, and ARHGEF9 depleted cells overexpressing ARHGEF9 cDNA. Scale bars 10 μm. (B) Graph depicted the number of FAs in all four conditions. (C) Histogram representing the orientation of single FAs.

We sought to rescue the FA phenotype resulting from ARHGEF9 custom-designed RNAi through overexpression of constitutively active ARHGEF9 cDNA (Methods). Cells were allowed to spread for 3h on fibronectin-coated glass bottom dishes before fixation and staining for Paxillin. We captured images using TIRF. Consistent with previous observations, depletion of ARHGEF9 resulted in fewer and rounder adhesions compared to control (Figure 7A). In contrast, overexpression of ARHGEF9 cDNA restored the number of FAs to that observed in wild-type cells (Figure 7B).

We also measured the alignment of the FAs with regard to the long axis of the cell. In control cells, most adhesions are oriented perpendicularly to the long axis of the cell. This reflects the fact that in spreading WM266-4 cells, large protrusions are made around the bulk of the cell periphery—leading to adhesions that form along the “sides” of cells (Figure 7A). In contrast, the bulk of FAs in ARHGEF9 depleted cells are oriented in parallel to the long axis and are localized to large protrusions. These structures are more typical of classic leading-edge protrusion (Figure 7A). Expression of ARHGEF9 cDNA in ARHGEF9 depleted cells rescues the defects in FA orientation and resulted in FAs that are localized radially around the cell periphery (Figures 7A and 7C). Thus, ARHGEF9 is required for both single FA morphogenesis, and overall, FA organization in WM266-4 cells.

**Figure 8:**
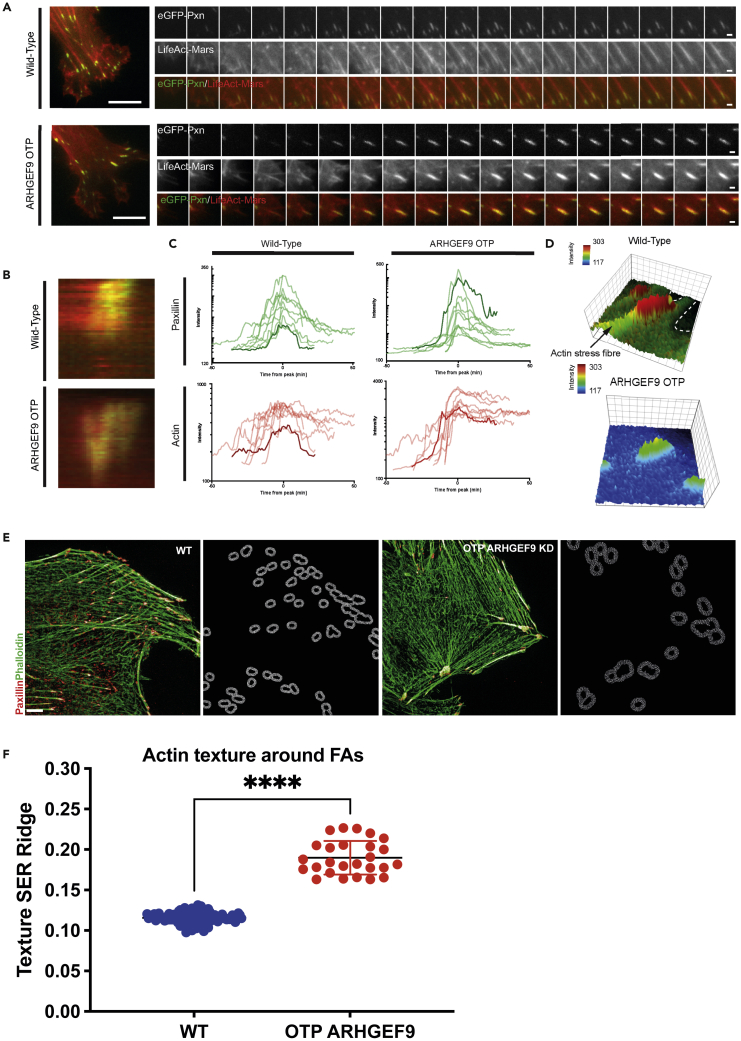
ARHGEF9 regulates actin dynamics at FAs (A) eGFP-Paxillin and LifeAct-Mars in Wild-Type and ARHGEF9 cells. Time series of adhesion formation at a protrusion in WT and ARHGEF9 OTP cells. (B and C) Paxillin and actin dynamics at an FA in wild-type and ARHGEF9 knockdown cells. Data produced from kymographs. Paxillin and actin dynamics at an FA in WT cells and OTP ARHGEF9 treated. (D) 3D surface maps of actin recruitment to FAs indicating the loss of stress fibers at FAs in OTP ARHGEF9 treated cells. (E) Representative SoRa microscopy images of F-actin structure and FAs. F-actin stained with phalloidin (green) and FA protein Vinculin (red). Actin texture features were measured around the FAs in the protrusions of control and ARHGEF9 depleted cells. Scale bars, 50μm. (F) There is significant difference in F-actin texture around single FAs between control (n = 107) and ARHGEF9 (n = 26) depleted cells. Comparison between the two groups was performed by unpaired t test with Prism7 software (∗∗∗∗p < 0.0001).

As wild-type WM266-4 cells protrude, nascent FAs at the leading edge quickly recruit actin stress fibers, forming focal complexes, which rapidly mature into larger FAs (Figure 8Α). Unlike wild-type cells, ARHGEF9 knockdown results in decreased stress fiber formation at nascent FAs in protrusions, though more mature adhesions in the cell body had thick, but shorter stress fibers compared to wild-type (Figure 8Α). This suggests a defect in actin recruitment to FAs in ARHGEF9 depleted cells. Thus, we conclude that ARHGEF9 is required for FAs morphogenesis in melanoma cells.

To investigate actin and FA dynamics we measured F-actin and Paxillin intensities using kymographic analysis over a region parallel to the long axis of the adhesion (Figure 8Β). We plotted the mean Paxillin and F-actin signal across the kymograph over time. We aligned the Paxillin and F-actin traces across multiple adhesions to the peak Paxillin intensity, capturing the most mature state of the adhesion. In control cells, we observed that peak Paxillin intensity followed increases in F-actin intensity (Figures 8C and 8D). These observations are consistent with the idea that actin polymerizes at the leading edge of filopodia structures which mark the site of FA formation. In WT cells, F-actin intensity remained relatively high even as Paxillin intensity decreased—reflecting stress fiber formation behind the FAs. However, in ARHGEF9 depleted cells, although Paxillin and F-actin were recruited to sites of ECM-adhesion, even to levels that exceeded that in WT cells, this occurred simultaneously with FA formation (Figure 8C). Thus, in WM266-4 cells, F-actin first polymerizes to mark the site of FA formation in an ARHGEF9-dependent manner but is also further polymerized at the adhesion independently of ARHGEF9. Following ARHGEF9 depletion, F-actin and Paxillin recruitment to protrusions occurs in parallel, and F-actin accumulation does not predict FA assembly.

To validate the idea that ARHGEF9 is required for actin polymerization that drives FA formation, we next quantified F-actin organization in 1-2 μm regions around single FAs in fixed cells. We described the organization of F-actin around each FA in WT and ARHGEF9 depleted cells using the “SER Ridge” texture—a feature describing regions that contain high-intensity pixels neighboring sharp drops in intensity (“ridges”) (Figure 8E). In control cells, the actin “SER Ridge” score is relatively low (approx. 0.12 a.u) which reflects the dense organization of F-actin filaments in wild-type cells. However, there is a median 1.6-fold increase in the SER Ridge score of F-actin around FAs in ARHGEF9 KD cells (Figure 8F). This reflects the observation that F-actin is more diffuse at FAs in ARHGEF9 depleted cells. Thus, ARHGEF9 depletion affects both FA morphogenesis and actin organization near FAs.

### ARHGEF9 is required for traction at adhesion

**Figure 9:**
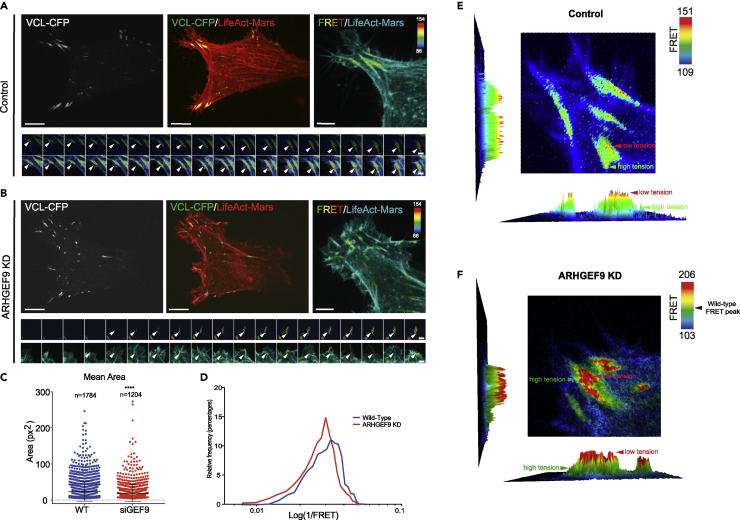
Vinculin tension sensor measuring vinculin dynamics in WM266-4 melanoma cells (A) Representative image of a WT cell transfected with LifeAct-Mars for actin (red) and Vinculin sensor (CFP) for its adhesions. (B) Representative image of an ARHGEF9 KD cell transfected with LifeAct-Mars for actin (red) and Vinculin sensor (CFP). (C) The mean area of Vinculin in FAs is less in comparison to the WT cells (∗∗∗∗p < 0.0001). (D) The FRET ratio for ARHGEF9 KD cells shows less tension compared to WT cells. (E and F) Representative images of FRET intensity show high and low tension on focal adhesions on WT and ARHGEF9 depleted cells.

Because ARHGEF9 plays a role in regulating FA dynamics, we next investigated the depletion of ARHGEF9 affecting the tensile force generated by FAs. To investigate whether ARHGEF9 depletion alters tension dynamics in WM266-4 cells, we used a Vinculin tension sensor (Vinc-TS) (Grashoff et al., 2010). When first recruited to nascent FAs, Vinc-TS exists in an inactive (closed) state. Under tension, cryptic Vinculin binding sites are revealed (Pasapera et al., 2010). At low tension and a closed conformation FRET occurs between mTeal (mTFP1) and Venus. Under tension, this FRET signal is lost. Using this reporter, we quantified changes in total Vinculin, and tension dynamics in control and ARHGEF9 knockdown cells (Figures 9A and 9B). Whilst adhesions forming in control cells have a low mean FRET signal (YFP transfer ratio), ARHGEF9 knockdown resulted in an increase in FRET signal (Figures 9C and 9D). This suggests that an increased proportion of the Vinculin reporter is in the closed conformation and thus under low tension. As the 1/YFP ratio is proportional to tension, ARHGEF9 resulted in a reduction in mean tension over an adhesions life (0.92-fold) as well as minimum tension (0.65-fold). Thus, ARHGEF9 is required to generate actomyosin tension on FAs.

### ARHGEF9 depleted cells invade less through soft collagen

**Figure 10:**
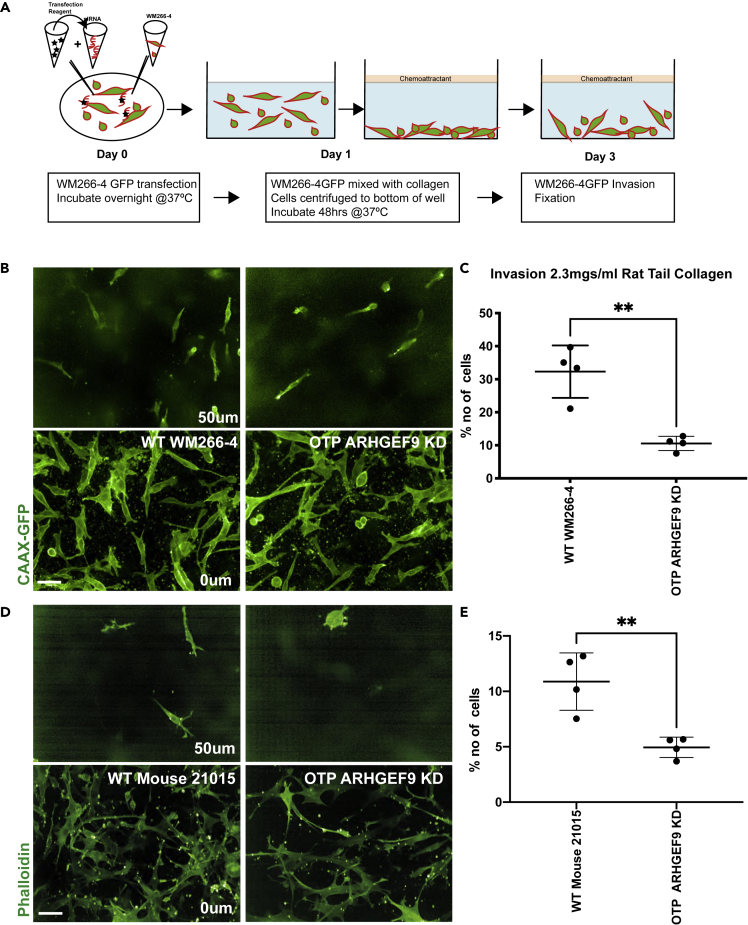
Human WM266-4 and mouse 21015 melanoma cells invading through thick collagen (A) Schematic representation of a “bottom up” invasion assay. (B) Representative images of WM266-4 showing thick collagen invasion assay at different depths. Scale bars, 50 μm. (C) Percentage of invasion index in 2.3 mgs/mL rat tail collagen indicated that ARHGEF9 KD cells invade less through thick collagen compared to WT WM266-4 cells. Comparison between the two groups was performed by unpaired t test with Prism7 software (∗∗p < 0.01). (D) Representative images of mouse 21015 showing thick collagen invasion assay at different depths. Scale bars, 50 μm. (E) Percentage of invasion index in 2.3 mgs/mL rat tail collagen indicated that ARHGEF9 KD cells invade less through thick collagen compared to WT mouse 21015 cells. Comparison between the two groups was performed by unpaired t test with Prism9 software (∗∗p < 0.01).

We depleted ARHGEF9 by siRNA in CAAX-GFP expressing WM266-4 cells and measured their invasion ability compared to control cells using a “bottom up” invasion assay. Briefly, we suspended control and ARHGEF9 depleted cells in 2.3 mgs/mL rat tail type I collagen (soft hydrogel), centrifuged the cells to the bottom of the gel, and then let the cells invade into the gel over 48h (Smith et al., 2008) (Figure 10A). Fluorescent imaging of the number of cells in each plane showed that control cells invaded upwards into the gel into depths as far as 60 μm (Figure 10B). For all conditions, we calculated an “invasion index” which summarizes the extent to which cells invade through multiple planes of 20 μm thick. In 48h, 33 percent (STDEV±1.6) of control cells invaded the gel, 10 percent into depths of 40 μm, and 2 percent into depths of 100 μm. In contrast, 27 percent (STDEV±3) of ARHGEF9 depleted cells invaded the gel, which represented a significant decrease compared to control (unpaired t-test; ∗∗p < 0.01). Only 8 percent (STDEV±1) of ARHGEF9 depleted cells invaded to 40 μm, and less than 1 percent invaded to 100 μm (Figure 10C). Moreover, ARHGEF9 depletion in mouse 21015 cells significantly blocked their ability to invade collagen matrices (unpaired t-test; ∗∗p < 0.01), (Figures 10D and 10E). Thus, ARHGEF9 is required for invasion.

## Discussion

WM266-4 cells, likely due in part to *PTEN* depletion (Yin et al., 2013), can generate transitory protrusions on soft 2D matrices which require non-canonical GTPases (Cooper et al., 2015). These protrusions appear to be stabilized when cells are embedded in soft 3D collagen and are rich in both filopodia and lamella-type structures (Figure 1). The stabilization of protrusion in 3D likely occurs as cells can form an increased number of integrin-based adhesions to the collagen fibers that surround them (Doyle et al., 2021).

Thus, in some cells, filopodia serve as a means by which cells can initially invade and attach to 3D spaces. Specifically, filopodial finger-like protrusions could allow cells to drill into matrices (Leijnse et al., 2022) and form attachments that coat the surface of the protrusion. Such attachments would allow cells to generate traction in 3D. In contrast, thin sheet-like lamellipodia are better suited to establishing attachments in 2D. We propose that ARHGEF9 is essential for filopodial formation at the establishment of FAs at sites of filopodia formation. ARHGEF9 has been observed to be recruited to neuronal cell membranes via interactions with neuronal factors such as Neuroligin-2 (Soykan et al., 2014; Poulopoulos et al., 2009). Based on this and our observation that ARHGEF9-GFP is localized to cytosolic, and membrane compartments we propose that ARHGEF9 principally acts at the cell membrane to activate CDC42 and promote rapid actin polymerization. These filopodia serve as “factories” for integrin cluster activation, attachment, and protrusion stabilization. Indeed, overexpression of ARHGEF9 promotes increased protrusion number and length. It is also striking that WM266-4 cells resemble neuronal cells where filopodia have been observed to drive adhesion in growth cones (Fischer et al., 2018). Thus, the effects of ARHGEF9 depletion on FA morphogenesis are likely to be a consequence of defects in actin polymerization dynamics.

Notably, on 2D and soft matrices ARHGEF9 depletion resulted in largely flat, poorly contractile cells and an inability to adopt ameboid forms. We propose that such defects are largely owing to a failure of ARHGEF9 depleted cells to promote rapid CDC42 polymerization in protrusions which act to engage FA-actin clutches and generate traction (Case and Waterman, 2015). In the absence of traction at FAs, cells cannot generate sufficient contractile forces in order to become round. This is in contrast to the idea that ARHGEF9 promotes rounding by other mechanisms, i.e. by the upregulation of RhoA to mediate cortical actomyosin activity. Moreover, such a model differs from that by which ARHGEF9-mediated contractility is required to upregulate cell-wide hydrodynamic forces required for round cells to form transitory large protrusions (Houk et al., 2012). If this was the case, we would expect ARHGEF9 overexpression to lead to increased rounding.

Mutations in the ARHGEF9 gene are associated with intellectual disability (Hines et al., 2022) and with numerous disorders including delayed motor development and epilepsy with facial dysmorphism (Alber et al., 2017). Such phenotypes are driven largely by ARHGEF9’s function in neuronal cells. For example, at the post-synaptic membrane ARHGEF9 is recruited to NRG2-mediated cell-cell adhesion sites where it promotes the clustering of inhibitory glycine and γ-amino-butyric acid type A (GABA_A_) receptors (Kilisch et al., 2020). Clustering is driven by ARHGEF9-mediated recruitment of cytoskeletal-associated protein Gephyrin, which reorganizes actin and inhibitory receptors. We propose that in melanoma cells, ARHGEF9 functions in a somewhat similar manner, where ARHGEF9 recruitment to the leading edge promotes actin reorganization into filopodia. Reorganized actin in filopodia serves to cluster and ultimately activate components of adhesion complexes. One outstanding question is which upstream factors may initiate the initial recruitment and activation of ARHGEF9.

The reliance of WM266-4 cells on ARHGEF9 and CDC42 for the regulation of cell shape suggests that the activation of this pathway, either at the transcriptional or post-translational levels, represents a means by which melanoma tumor cells evolve the ability to metastasize. By upregulating factors such as these which promote filopodia formation and concomitant adhesion engagement, this may allow metastatic cells greater ability to explore 3D spaces with varying geometry and rigidity. Indeed, the activation of this pathway may represent a case where melanoma cells “borrow” mechanisms used by related cell types such as endothelial tip cells or neurons, to perform certain tasks. This finding thus presents therapeutic opportunities to target metastasis.

### Limitations of the study

In our study, we showed that ARHGEF9, via its GEF activity, promotes the formation of actin-rich filopodia which serves to establish and stabilize focal adhesions. Although this idea is consistent with findings from numerous studies that have shown that ARHGEF9 activates CDC42 (Xiang et al., 2006; Harvey et al., 2004; Tyagarajan et al., 2011) we cannot exclude the possibility that ARHGEF9 regulates actin and adhesions via different GTPases. Further studies are necessary to elucidate specific molecular mechanisms by which ARHGEF9 regulates morphogenesis.

## STAR★Methods

### Key resources table

**Table:** 

REAGENT or RESOURCE	SOURCE	IDENTIFIER
**Antibodies**		

Purified mouse Anti-Paxillin	BD Biosciences	CAT#610052
Anti-Vinculin mAB produced in mouse	Sigma	CAT#V4505: RRID: AB_477617
Phospho-Paxillin (Tyr31)	Invitrogen	CAT#44-720G: RRID: AB_2533732
anti-GAPDH mouse mAB	Novus Biotechne	CAT#NB300-221
Purified rabbit Anti-Paxillin	Abcam	CAT# ab32084:RRID: AB_779033
Rabbit anti-phospho (Y397) FAK	Abcam	CAT#Ab81298:RRID: AB_1640500
Alexa Fluor® 488 goat anti mouse IgG	Invitrogen	CAT#A11029:RRID: AB_2534088
Alexa Fluor™ 488 goat anti rabbit IgG	Invitrogen	CAT#A11034RRID: AB_2576217
Alexa Fluor™ 647 goat anti mouse IgG	Invitrogen	CAT#A21235:RRID: AB_2535804
Alexa Fluor™ 647 F(ab')2 goat anti rabbit IgG	Invitrogen	CAT#A21246:RRID: AB_2535814
Rabbit anti- ARHGEF9 antibody	St Johns Labs	CAT# STJ110271

**Chemicals, peptides, and recombinant proteins**		

Alexa Fluor® 488 Phalloidin	Invitrogen	CAT#A12379
Phalloidin-Atto 647N	Sigma	CAT#65906
CellTracker™ Orange CMTMR Dye	ThermoFischer	CAT#C2927
Hoescht	Invitrogen	CAT#H3569
DMEM-Dulbecco’s Modiefield Eagle Medium	ThermoFischer	CAT#41966029
Leibovitz’s L-15 Medium, no phenol red	ThermoFischer	CAT#21083027
Trypsin-EDTA (0.25%), phenol red	ThermoFischer	CAT#25200056
Penicillin-Streptomycin (10,000 U/mL)	ThermoFischer	CAT#15140122
Lipofectamine™ RNAiMAX Transfection Reagent	ThermoFischer	CAT#13778500
Effectene Transfection Reagent	Qiagen	CAT#301425
Fetal Bovine Serum, qualified, heat inactivated	ThermoFischer	CAT#16140071
Triton™ X-100	Sigma	CAT#X100
Pierce™ 16% Formaldehyde (w/v), Methanol-free	ThermoFischer	CAT#28908
Corning® Collagen I, Rat Tail, 100mg	Corning	CAT#354236
BSA	Sigma	CAT#A2153
16% formaldehyde solution	ThermoFischer	CAT#28908
Blebbistatin	Sigma	CAT# B0560
FAK inhibitor	Tocris	CAT#3239
Y-27632	Sigma	CAT#Y0503
Fibronectin human plasma	Sigma	CAT#F0895

**Critical commercial assays**		

Rneasy mini Kit	Qiagen	CAT#74904
HiSpeed Plasmid Maxi Kit	Qiagen	CAT#12663
ABI High-capacity RNA-to-cDNA	Applied Biosystems	CAT#4387406

**Experimental models: Cell lines**		

Human WM266-4	kind gift from the Marshall lab, ICR	N/A
Human WM266-4 eGFP CAAX	kind gift from the Marshall lab, ICR	N/A
Mouse 21015	kind gift from Malin Pendersen, ICR	N/A

**Oligonucleotides**		

ARHGEF9 Primers 5′GCCAGATCGACGATGAGGA-3′ 5′-TCTGGTCCCGGTTCTGTAGT-3′	SIGMA	N/A
ARHGEF9 Primers 5′-GGTTTCCTGCCAGCTTTGTG-3′ 5′-GCAGTCTGAATTGGGGTCCA-3′	SIGMA	N/A
GAPDH Primers 5′-AGATCCCTCCAAAATCAAG-3′ 5′-GGCAGATGATGACCCTTTT-3′	SIGMA	N/A
custom siRNA: ARHGEF9; GACUUGUAUUAUAAGUCAAGG, CCUUGACUUAUAAUACAAGUCUU	Dharmacon	N/A
On-TARGETplus Smart Pool custom libraries	Dharmacon	Table S1
siGENOME Smart Pool custom libraries	Dharmacon	Table S2
ON-Traget plus pool ARHGEF9	Dharmacon	L-020314-00-0005
siGENOME plus pool ARHGEF9	Dharmacon	M-020314-01-0005
Mouse ON-Traget plus pool ARHGEF9	Dharmacon	L-059568-01-0005

**Recombinant DNA**		

eGFP-Paxillin	kind gift from Chris Turner	
Talin-mApple	addgene	CAT# 54951
LifeAct-Mars	kind gift from the Calvo Lab	
cDNA ARHGEF9 human	Origene	RG230127
T61A ARHGEF9 human	(Reddy-Alla et al., 2010)	https://www.ncbi.nlm.nih.gov/pubmed/20345913

**Software and algorithms**		

Columbus Image Data Storage and Analysis System	PerkinElmer	http://www.cambridgesoft.com/ensemble/spotfire/Columbus/default.aspx
Slidebook software	3i Intelligent imaging	https://www.intelligentimaging.com
Prism	GraphPad	https://www.graphpad.com/scientific-software/prism/
Excel	Microsoft	
ImageJ	ImageJ	https://imagej.nih.gov/ij/
FAAS	(Berginski and Gomez, 2013)	https://faas.bme.unc.edu/
Primer3	Primer3	https://primer3.ut.ee/

### Resource availability

#### Lead contact

Further information and requests for resources and reagents should be directed to and will be fulfilled by the lead contact Chris Bakal (Chris.Bakal@icr.ac.uk) and Vicky Bousgouni (Vicky.Bousgouni@icr.ac.uk).

#### Materials availability

This study did not generate new materials or reagents.

### Experimental model and subject details

#### Cell culture

WM266-4 human and mouse 21015 melanoma cells were maintained in Dulbecco’s Modified Eagle Medium (DMEM) supplemented with 1% penicillin-streptomycin (ThemoFicher) and 10% FBS (ThermoFischer). The cultured cells were maintained at 5% CO_2_ in humidified chamber at 37°C.

Both cell lines were confirmed to be mycoplasma negative (e-Myco plus Mycoplasma PCR detection kit, iNTRON Biotechnology). Passaging was carried out using 0.25% Trypsin-EDTA (ThermoFischer) and resuspension in complete medium. Cell counting was performed using Countess automated cell counter with trypan blue exclusion (ThermoFischer).

### Methods details

#### Study design for screens on top of soft and stiff materials

An siGENOME pool library (Dharmacon) and OnTARGET*Plus* pool library (Dharmacon) were used for all screens. The siRNAs from each library were arrayed into 96 well plates for screens on top of soft collagen, hydrogel and into 384 well plates for screens on top of stiff plastic. Duplicate plates were used for both screens. Each plate contained two technical replicates per siRNA. Control wells were also included in each plate and were distributed in random wells across the plates.

#### High throughput RNAi screening on top of soft material

WM266-4 melanoma cells were pre labeled with 2μM CellTracker Orange CMTMR dye (ThermoFischer) as per manufacturer’s protocol. RNAi screens were performed in 96 well plates to which 160 nL/well siRNA (20 μM) were plated using an Echo liquid handler (LabCyte). Prior to seeding cells, 20 μL of OptiMEM (Invitrogen) containing 160 nL/well Lipofectamine RNAiMAX (Invitrogen) was added using a Multidrop Combi Reagent Dispenser (ThermoFischer) and plates were incubated for 30 min at room temperature (RT). One day after transfection cells were seeded on top of 100 μL of 1.8 mg/mL rat-tail collagen in DMEM plus 10% heat inactivated FBS prepared as per manufacturer protocol. Collagen was pre-aliquoted into wells of a 96-well, glass-bottomed, collagen-coated plates (PerkinElmer). After 24h of incubation cells were fixed by adding 100 μL of pre-warmed 8% methanol free formaldehyde (ThermoFischer) containing 5 μg/mL Hoescht stain (Invitrogen) and incubated for 1h at RT. Cells were imaged in the OPERA QEHS (PerkinElmer), with a 20x air immersion objective. Imaging was performed at a single timepoint capturing 15 wells per field and 200-plane z-stacks. Maximum intensity projections were used for image analysis.

#### High throughput RNAi screening on top of stiff material

RNAi screens were performed in 384-well Cell Carrier plates (PerkinElmer) to which 40 nL/well siRNA (20 μM) were plated using an Echo liquid handler (LabCyte). Prior to seeding cells, 10 μL of OptiMEM (Invitrogen) containing 40 nL/well Lipofectamine RNAiMAX (Invitrogen) was added using a Multidrop Combi Reagent Dispenser (ThermoFischer) and plates were incubated for 30 min at RT. For all wells containing siRNA and a subset of control wells, 5000 cells/well were seeded in 20 μL of complete medium. After 48h cells were fixed by adding 30 μL of pre-warmed 8% PFA (methanol free) (ThermoFischer), and incubated for 15 min at RT. After washing 3X with PBS, cells were permeabilized in 0.2% Triton X-100. Cells were blocked for 1h at RT with 0.5% BSA in PBS. After washing 3X with PBS, primary antibodies were added in 10 μL block solution, and plates were sealed and incubated overnight at 4°C. Following 3X washes in PBS, secondary antibodies were added and incubated for 2h at RT. Plates were washed 2X in PBS, incubated for 15 min with 5 μg/mL Hoechst, washed 1X with PBS, filled with 50 μL PBS, and sealed for imaging. Cells were imaged in the OPERA QEHS (PerkinElmer), with a 20x air immersion objective, on single plane.

#### Features and data processing

Automated segmentation was performed using Columbus software (PerkinElmer). In images from screens on top of soft collagen nuclei were segmented using the Hoechst channel and cell bodies were segmented using the CellTracker Orange channel. Morphological and texture features were extracted for all cells while cells touching the edge were removed. These features are the ‘signature’ of each cell and were used to train classifiers to categorize cells into three different distinct shape groups.

In images from screens on top of plastic nuclei were segmented using the Hoechst channel and cell bodies were segmented using the tubulin channel. Morphological and texture features were extracted for all cell while cells touching the edge were removed. Cells were categorized into three different distinct shape groups.

In all screens data from each plate was normalized against the whole plate and hits were determined for each cell shape. An siRNA was considered a hit when it enhanced or suppressed a specific phenotype across replicates. We calculated z-scores and set a threshold of Z = +/− 1.5 for a gene to be a hit.

#### Scanning electron microscopy experiments

On Day 0, control WM266-4 were transfected with control and OnTArget*Plus* SMARTpools ARHGEF9 (Dharmacon cat # L-020314-00-0005) using RNAimax (Invitrogen) and incubated for 24h. On Day 0, ARHGEF9 and control WM266-4 cells were transfected using RNAiMAX (Invitrogen) according to the manufacturer’s instructions and incubated for 24h. On Day 1, cells were transferred on 5 mm coverslips with a thick layer of 3 mgs/mL collagen. Coverslips were incubated for 24h before fixation. Coverslips were then fixed in 2.5% glutaraldehyde in PBS for 2h at room temperature, rinsed and stored in PBS. Samples were then dehydrated through a graded series of ethanol:water solutions (30%, 50%, 70%, 100% ethanol), 15 min in each. Coverslips were then transferred into a 50:50 mixture of 100% ethanol and Hexametthyldisilazane (HMDS) for 30 min, followed by 15 min in fresh HMDS. After 15 min most of the HMDS is removed and the remainder left to evaporate to dryness overnight in a fume hood. On the next day the now dry coverslips were mounted on Aluminum SEM stubs and taken to the Natural History Museum for sputter coating (Cressington 208 HR) and imaging on SEM (Zeiss Ultraplus FEGSEM).

#### Lattice lightsheet microscopy

WM266-4 melanoma cells expressing CAAX-GFP were embedded in 3 mgs/mL rat tail type I collagen on top of 5 mm glass coverslips pre coated with fibronectin 10 μg/mL. Coverslips with collagen and cells embedded were left in a well of a six well plate with 2 mL complete medium overnight in a tissue culture incubator. Prior to imaging the medium was changed in to pre warmed Leibovitz’s L-15 medium, phenol free. Coverslips were imaged using a Lattice Lightsheet Microscope (3i Intelligent Imaging).

#### Verification of ARHGEF9 mRNA depletion

ARHGEF9 OTP transfected and control cells were lysed with phenol:chloroform (TRIzol®,) and RNA was extracted with RNAeasy kit (Qiagen) according to manufacturer’s protocol. RNA was converted to cDNA with the ABI High-capacity RNA-to-cDNA conversion kit (Applied Biosystems) as per manufacturer’s protocol. ARHGEF9 Exon spanning primers were designed for qRT-PCR using the Primer3 package:

ARHGEF9: Forward 5′GCCAGATCGACGATGAGGA-3′

Reverse 5′-TCTGGTCCCGGTTCTGTAGT-3′

ARHGEF9: Forward 5′-GGTTTCCTGCCAGCTTTGTG-3′

Reverse 5′-GCAGTCTGAATTGGGGTCCA-3′

GAPDH: Forward 5′-AGATCCCTCCAAAATCAAG-3′

Reverse 5′-GGCAGATGATGACCCTTTT-3′

Quantitative real-time PCR was conducted using the Invitrogen SyBR green master mix with fluorescence amplification being measured using the QuantStudio six Flex Real-Time PCR System (Thermo).

Fold changes in gene expression were calculated according to the 2-ΔΔCT method and sample measurements normalised against amplification from GAPDH control.

#### Western blots for OTP ARHGEF9 KD verification

Western blot lysis was conducted using a urea buffer (8 M Urea, 250 mM NaCl, 40 mM HEPES pH7.9, 5% glycerol, protease and phosphatase inhibitor cocktail (Thermo)). Cells were lysed on ice withcell scrapers used to harvest cells. All cell lysates were sonicated at amplitude of 10 μm (SoniPrep150). A Laemmli running buffer (2% SDS, 175mM DTT, 0.002% Bromophenol blue, 0.063mM Tris 6.8 pH, 10% Glycerol) was added to samples and samples were run on a Novex™ 4-20% Tris-Glycine Mini Gels using Novex™ SDS Tris-Glycine running buffer. Samples were initially run at 110V through the stacking gel then at 150V through running gel for a total of 1.5h. Bands were transferred to an Immobilon®-FL transfer membrane at 4°C in transfer buffer (Glycine 192 mM, Tris 25 mM, Methanol 200 mM). Afterwards, transfer membranes were blocked in 5% dry non-fat milk in TBS-T for 1h at room temperature. Transfer membranes were incubated in primary antibodies at 4°C overnight. Rabbit anti- ARHGEF9 antibody was used at a 1:500 dilution (5% milk) and mouse anti-GAPDH 1:2000 (5% milk). Dylight 800 nm anti-rabbit and Dylight 600nm anti-mouse antibodies (1:15,000) were used to visualise proteins using an Odyssey Li-Cor Imaging system.

#### Experiments for focal adhesions dynamics

To study the effects of different gene knockdowns on focal adhesion and cytoskeletal dynamics, we co-transfected WM266-4 melanoma cells with different combinations of plasmid reporters and siRNAs. Cells were seeded on a 6-well plate, 24h prior to transfection at a density of 1 × 10^6^ cells/well. Effectene (Qiagen) transfection mixtures contained 1 μg of total plasmid, 2.56 μL of siRNA, 97μL of EC buffer, 8 μL of enhancer and 5 μL of Effectene – a total volume of 113 μL of transfection mixture. Prior to the addition of transfection mixture, 1.6 mL of fresh complete DMEM was added per well. A final concentration of 25 μM of siRNA was used. Cells were incubated for 48h (unless otherwise indicated).

#### Recombinant DNA

eGFP-Paxillin reporter construct from Chris Turner was used to visualise adhesion dynamics. Actin was visualised using LifeAct-Mars (Riedl et al., 2008). ARHGEF9 overexpression was conducted using an OriGene ARHGEF9-GFP construct. The vinculin Tension sensor was made by Martin Schwartz group (Grashoff et al., 2010). Prior to use, 1 μg of plasmid was used to transform 50 μL of DH5α competent *E. coli*. After transformation by heat shock, bacteria were incubated in LB broth for 1h at 37°C then spread over a plate and incubated overnight. Single colonies were picked from each plate and used to generate 150 mL overnight cultures. Plates and LB broth cultures contained ampicillin to select for ampicillin resistance conferred by eGFP-Paxillin construct. After 24h cultures were centrifuged at 6000xg and plasmid DNA extracted using the Qiagen Maxi Prep Kit. Extracted DNA was quantified using a NanoDrop 1000 spectrophotometer (ThermoFischer).

#### Live total internal reflection fluorescence microscopy

48h after transfection cells were harvested by trypsinisation and ∼200,000 cells were transferred to a 35 mm Collagen Coated-MatTek glass-bottom dish. Cells were imaged in phenol-free DMEM (10% FBS 5% Pen-Strep) with 25mM HEPES to buffer for any variations in CO2 levels. MatTek dishes were incubated at 37°C for 1h before imaging allowing cells to adhere to collagen surface. Imaging was conducted between 1 and 8h after plating. Cells were imaged on a 3i microscopy vector total internal reflection (TIRF) microscope system (3i Intelligent Imaging). Incubation during imaging was maintained by an OkoLabs climatisation chamber at 37°C. eGFP-Paxillin images were captured using a 63x oil immersion objective at an exposure of 200 ms. Cells were imaged at an interval of 60 s over a minimum period of 40 min. Images were then analyzed using the focal adhesion analysis server (FAAS) (Berginski et al., 2011).

#### Small molecule inhibitors

Three small molecule inhibitors were used in this work in order to perturb adhesion dynamics: Y-27632 (2μM), PF-573288 (10μM), and Blebbistatin (10μM). These drugs were added to cells as they spread on collagen (Sero and Bakal, 2017). Fluorescence Resonance Energy Transfer (FRET) Vinculin Tension Sensor FRET experiments were conducted on a 3i spinning disc confocal using a 63x oil-immersion objective at an exposure of 200 ms. Cells were imaged at an interval of 60 s over a minimum period of 40 min. FRET transmission was calculated by measuring the ratio of CFP donor emission to YFP acceptor emission. Images were then analyzed using FAAS.

#### Focal adhesion dynamics analysis

Live cell tracking of focal adhesions was conducted using the FAAS. This open-source MATLAB based script allowed the segmentation and tracking of focal adhesion signal, outputting both static properties of adhesions over time, such as major axial length and area, as well as dynamic properties such as assembly rates. For all properties examined in this paper, a single focal adhesion had to be tracked over five frames to be included in this dataset. Furthermore, dynamics models, based on a log-linear regression, had to have an R2 >0.9.

#### Plasmid transfections

WT ARHGEF9 cDNA plasmid was purchased from OriGene (cat: RG230127) and ARHGEF9 Mutant plasmid was a kind gift from Papadopoulos T., Max-Planck Institute for Brain Research. Cells were transfected using the Effectene Transfection kit (Qiagen) according to manufacturer’s protocol. Prior to transfections cells were seeded 1.0 × 10^6^ cells/mL in 2mL medium per well and incubated overnight. Experiments were performed 48h after transfections.

#### Protrusions rescue experiment for WM266-4 cells embedded in collagen

On Day 0, control WM266-4 were transfected with control and custom-designed siRNA ARHGEF9, (cARHGEF9) (Dharmacon custom desιgned # GACUUGUAUUAUAAGUCAAGG, CCUUGACUUAUAAUACAAGUCUU) using RNAimax (Invitrogen) and incubated for 24h. On day 1 cells were transfected with ARHGEF9 cDNA and ARHGEF9 T61A mutant using the Effectene Transfection kit (Qiagen) according to manufacturer’s protocol. On day 2 after transfection 10^5^ cells/mL were re-suspended in 500 μL of 2 mgs/mL collagen rat tail. A 100 μL of collagen and cells mix were dispensed in five wells per condition onto 96 well view plates (PerkinElmer). After 24h of incubation cells were fixed with pre-warmed 4% PFA (methanol free) (ThermoFischer) for 20 min at RT and stained for F-actin using Phalloidin Alexa 647 and Hoescht. Cells were imaged with a 20X air lens at different planes.

#### Focal adhesions rescue experiment for WM266-4 cells

On Day 0, control WM266-4 were transfected with control and custom-designed siRNA ARHGEF9, (cARHGEF9) (Dharmacon custom desιgned # GACUUGUAUUAUAAGUCAAGG, CCUUGACUUAUAAUACAAGUCUU) using RNAimax (Invitrogen) and incubated for 24h. On day 1 cells were transfected with ARHGEF9 cDNA using the Effectene Transfection kit (Qiagen) according to manufacturer’s protocol. On day 2 cells were trypsinised and re-suspended in complete medium. Approximately, 10,000 cells were transferred on MatTek dishes pre coated with fibronectin 10 μg/mL, in 2 mL DMEM and left to spread for approximately 3h. Cells were fixed with pre-warmed 4% PFA (methanol free) (ThermoFischer) for 15 min at RT. After washing 3X with PBS, cells were permeabilized in 0.2% Triton X-100 and incubated at RT for 10 min. Cells were then washed in PBS before being blocked in 0.2% bovine serum albumen, for 1h. Primary antibody, rabbit anti-Paxillin (1:500), was diluted in block and incubated at room temperature for 1.5h. After primary, samples were washed, and secondary antibody Alexa 647 was diluted in block and incubated on samples for 2h (1:1000). Samples were imaged in PBS. Images were captured with a 63x oil immersion objective at a single time point. We set 10 pixels as threshold of minimum size of an adhesion and single FAs were segmented and counted using that FAAS: 451 adhesions in WT cells, 406 adhesions in WT cells overexpressing WT ARHGEF9, 174 adhesions in ARHGEF9 KD cells and 419 adhesions in ARHGEF9 KD cells overexpressing WT ARHGEF9 (n = 3 per condition).

#### Actin texture around adhesions imaged with super-resolution via optical re-assignment

WM266-4 melanoma cells were reverse transfected with OnTArget*Plus* SMARTpool ARHGEF9 and Wild Type cells (Dharmacon cat # L-020314-00-0005), at a concentration of 20 μM in a six well plate. Transfections were carried out using Lipofectamine RNAiMAX (Invitrogen) according to the manufacturer’s instructions. 48h later cells were trypsinised and re-suspended in DMEM. Approximately, 10,000 cells were transferred on MatTek dishes pre coated with fibronectin 10 μg/mL, in 2 mL DMEM. Cells were left to spread overnight at 37°C in a tissue culture incubator. Next day cells were fixed with pre-warmed 4% PFA (methanol free) (ThermoFischer) for 15 min at RT. After washing 3X with PBS, cells were permeabilized in 0.2% Triton X-100 and incubated at RT for 10 min. Cells were then washed in PBS before being blocked in 0.2% bovine serum albumen, for 1h. Primary antibody, rabbit anti-Paxillin (1:500), was diluted in block and incubated at room temperature for 1.5h. After primary, samples were washed, and secondary antibody Alexa 647 and Phalloidin 488 were diluted in block and incubated on samples for 2h (1:1000). Samples were imaged in PBS. Super-resolution images were captured with a 63x oil immersion objective at a single time point (SoRa, 3i). Images were imported into Columbus software (PerkinElmer) and actin texture was measured around a ring region area of single adhesions.

#### Fluorescence resonance energy transfer (FRET) vinculin tension sensor

FRET experiments were conducted on a 3i spinning disc confocal using a 63x oil immersion objective at an exposure of 200 ms. Cells were imaged at an interval of 60 s over a minimum period of 40 min. FRET transmission was calculated by measuring the ratio of CFP donor emission to YFP acceptor emission. Images were then analyzed using the FAAS.

#### 3D invasion assays

WM266-4 melanoma cells expressing CAAX GFP were reverse transfected with OnTArget*Plus* SMARTpool ARHGEF9 (Dharmacon cat # L-020314-00-0005) at a concentration of 20 μM in a six well plate. Transfections were carried out using Lipofectamine RNAimax (Invitrogen) according to the manufacturer’s instructions. On second day after transfection 10^5^cells/mL were re-suspended in 500 μL of 2.3 mgs/mL collagen rat tail. A 100 μL of collagen and cells mix were dispensed in quadruplicate wells onto 96 well view plates (PerkinElmer). Plates were centrifuged @ 1200 rpm for 5 min at 4°C and incubated in a tissue culture incubator. After 48h of incubation cells were fixed with 4% PFA (methanol free) (ThermoFischer) containing 5 μg/mL Hoescht stain. Following 3X washes in PBS, 100 μL of Phalloidin 488 was added per well. Plates were imaged using an Opera QEHS (PerkinElmer) using four planes and taking 31 fields of view/well. To calculate the invasion index, the sum of cells was calculated at three different planes, 30, 60 and 90 μm and divided by total number of cells.

Mouse melanoma cells 21015 were obtained from Malin Pendersen, The Institute of Cancer Research. WT were reverse transfected with OnTArget*Plus* SMARTpool ARHGEF9 (Dharmacon cat # L-059568-01-0005) at a concentration of 20 μM in a six well plate. Transfections were carried out using Lipofectamine RNAimax (Invitrogen) according to the manufacturer’s instructions. On second day after transfection 10^5^ cells/mL were re-suspended in 500 μL of 2.3 mgs/mL collagen rat tail. A 100 μL of collagen and cells mix were dispensed in quadruplicate wells onto 96 well view plates (PerkinElmer). Plates were centrifuged @ 1200 rpm for 5 min at 4°C and incubated in a tissue culture incubator. After 48h of incubation cells were fixed with 4% PFA (methanol free) (Thermo) containing five ug/mL Hoescht stain. Following 3X washes in PBS, 100 μL of Phalloidin 488 was added per well. Plate was imaged using an Opera QEHS (PerkinElmer) using four planes and taking 25 fields of view/well. To calculate the invasion index, the sum of cells was calculated at three different planes, 30, 60 and 90 μm and divided by total number of cells.

### Quantification and statistical analysis

The ranking list of the siRNAs and determination of hit genes was derived from the calculation of the Z′-factor. Quantification was represented as the mean ± SD. Statistical significance (p value) for all experiments was assessed using, two-way ANOVA or Kolmogorov-Smirnov test (multiple groups) and unpaired t-test (two groups) in the software GraphPad Prism 7 to 9. The exact number of replicates is reported in the figures and the figure legends. All statistical calculations are included in the figures and p values less than 0.01 were accepted as significant, indicated by asterisks.

## Data Availability

The data reported in this paper will be shared by Chris Bakal (Chris.Bakal@icr.ac.uk) and Vicky Bousgouni (Vicky.Bousgouni@icr.ac.uk). This paper does not report original code. Any additional information required to reanalyze the data reported in this paper is available from the lead contact upon request.

## Supplemental information

Document S1. Figures S1 and S2, Tables S3-S8

Table S1. On-TARGETplus Smart Pool custom libraries related to STAR methods

Table S2. siGENOME Smart Pool custom libraries related to STAR methods
