# Supplementary material for: ARHGEF9 regulates melanoma morphogenesis in environments with diverse geometry and elasticity by promoting filopodial-driven adhesion
Source: iScience. 2022 Aug 8;25(8):104795. doi: 10.1016/j.isci.2022.104795 (PMC9418690; doi:10.1016/j.isci.2022.104795)
Supplement: Document S1. Figures S1 and S2, Tables S3-S8 [file mmc1.pdf]

iScience, Volume *25*

Supplemental information

ARHGEF9 regulates melanoma morphogenesis in environments with diverse geometry and elasticity by promoting filopodial-driven adhesion

Vicky Bousgouni, Oliver Inge, David Robertson, Ian Jones, Innes Clatworthy, and Chris Bakal

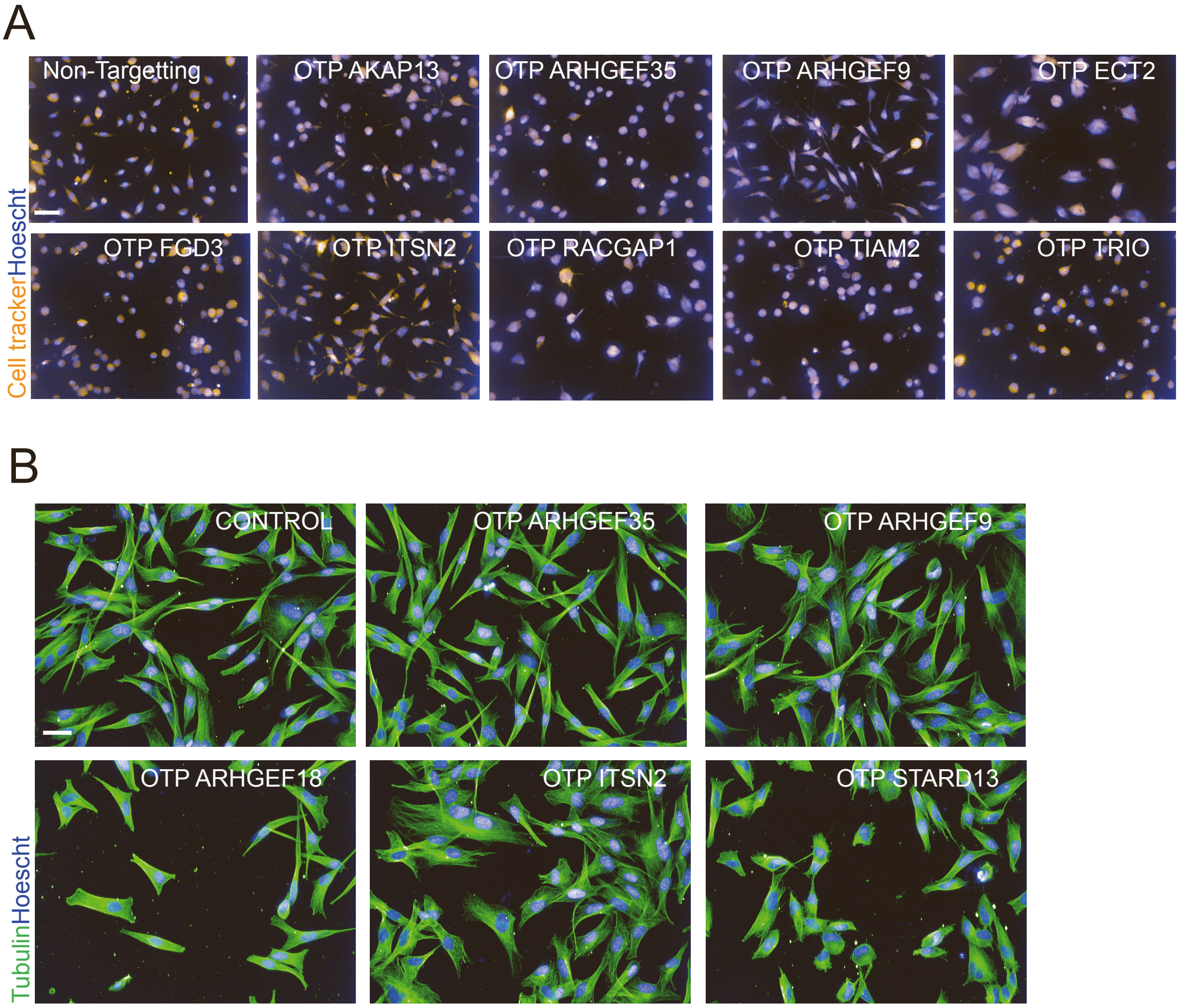

Figure S1 related to figures 2E and 3E: Candidate siRNAs depletion in human WM266-4 cells
(A) Representative images of cells cultured on top of soft collagen. Scale bars, 50 μm.
(B) Representative images of cells cultured on top of plastic. Scale bars, 50 μm.

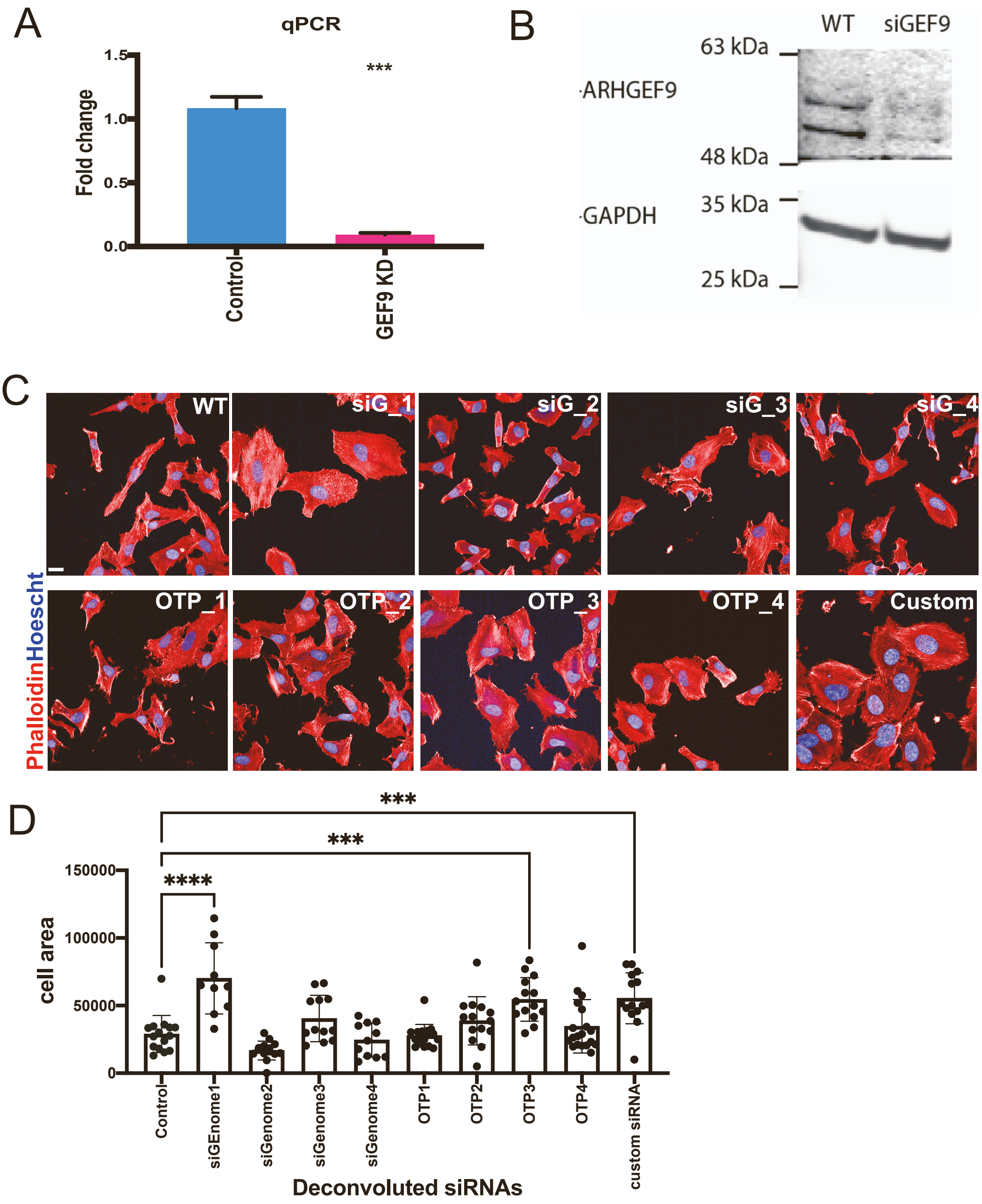

Figure S2 related to figures 2 and 3:Verification of mRNA depletion
(A) Graph depicting the fold change in the expression of ARHGEF9 in ARHGEF9 depleted and WT cells. ARHGEF9 is significantly down regulated compared to WT (*** $p < 0.001$).
(B) Western Blot indicating downregulation of ARHGEF9 in ARHGEF9 depleted cells in comparison to WT.
(C) Representative images of WM266-4 transfected with deconvoluted siGENOME ARHGEF9 siRNAs, deconvoluted OnTARGET*plus* Pool siRNAs and custom designed ARHGEF9 siRNA. Scale bars 100 μm
(D) Graph depicting single cells area per condition.

cells Selected - Number of Objects
cells Selected - Intensity Nucleus Exp1Cam1 Mean - Mean per Well
cells Selected - Intensity Nucleus Exp1Cam1 Mean - StdDev per Well
cells Selected - Intensity Cytoplasm Exp1Cam2 Mean - Mean per Well
cells Selected - Intensity Cytoplasm Exp1Cam2 Mean - StdDev per Well
cells Selected - Intensity Cell Exp1Cam2 Mean - Mean per Well
cells Selected - Intensity Cell Exp1Cam2 Mean - StdDev per Well
cells Selected - Nucleus Area [ÂµmÂ²] - Mean per Well
cells Selected - Nucleus Area [ÂµmÂ²] - StdDev per Well
cells Selected - Nucleus Roundness - Mean per Well
cells Selected - Nucleus Roundness - StdDev per Well
cells Selected - Cell Area [ÂµmÂ²] - Mean per Well
cells Selected - Cell Area [ÂµmÂ²] - StdDev per Well
cells Selected - Cell Roundness - Mean per Well
cells Selected - Cell Roundness - StdDev per Well
cells Selected - Cell Symmetry 02 - Mean per Well
cells Selected - Cell Symmetry 02 - StdDev per Well
cells Selected - Cell Symmetry 03 - Mean per Well
cells Selected - Cell Symmetry 03 - StdDev per Well
cells Selected - Cell Symmetry 04 - Mean per Well
cells Selected - Cell Symmetry 04 - StdDev per Well
cells Selected - Cell Symmetry 05 - Mean per Well
cells Selected - Cell Symmetry 05 - StdDev per Well
cells Selected - Cell Symmetry 12 - Mean per Well
cells Selected - Cell Symmetry 12 - StdDev per Well
cells Selected - Cell Symmetry 13 - Mean per Well
cells Selected - Cell Symmetry 13 - StdDev per Well
cells Selected - Cell Symmetry 14 - Mean per Well
cells Selected - Cell Symmetry 14 - StdDev per Well
cells Selected - Cell Symmetry 15 - Mean per Well
cells Selected - Cell Symmetry 15 - StdDev per Well
cells Selected - Cell Threshold Compactness 30% - Mean per Well
cells Selected - Cell Threshold Compactness 30% - StdDev per Well
cells Selected - Cell Threshold Compactness 40% - Mean per Well
cells Selected - Cell Threshold Compactness 40% - StdDev per Well
cells Selected - Cell Threshold Compactness 50% - Mean per Well
cells Selected - Cell Threshold Compactness 50% - StdDev per Well
cells Selected - Cell Threshold Compactness 60% - Mean per Well
cells Selected - Cell Threshold Compactness 60% - StdDev per Well

cells Selected - Cell Axial Small Length - Mean per Well
cells Selected - Cell Axial Small Length - StdDev per Well
cells Selected - Cell Axial Length Ratio - Mean per Well
cells Selected - Cell Axial Length Ratio - StdDev per Well
cells Selected - Cell Radial Mean - Mean per Well
cells Selected - Cell Radial Mean - StdDev per Well
cells Selected - Cell Radial Relative Deviation - Mean per Well
cells Selected - Cell Radial Relative Deviation - StdDev per Well
cells Selected - Cell Profile 1/5 - Mean per Well
cells Selected - Cell Profile 1/5 - StdDev per Well
cells Selected - Cell Profile 2/5 - Mean per Well
cells Selected - Cell Profile 2/5 - StdDev per Well
cells Selected - Cell Profile 3/5 - Mean per Well
cells Selected - Cell Profile 3/5 - StdDev per Well
cells Selected - Cell Profile 4/5 - Mean per Well
cells Selected - Cell Profile 4/5 - StdDev per Well
cells Selected - Cell Profile 5/5 - Mean per Well
cells Selected - Cell Profile 5/5 - StdDev per Well

Table S3 related to figure 2: Morphological and texture features, Screens on top of soft collagen.

Gene	Z Score Round	Gene	Z Score Spindle	Gene	Z score Star
ECT2	-2.1	FGD3	-1.7	FGD3	-2.0
FAM13A1	-1.9	ARHGAP20	-1.7	ARHGEF5	-1.8
RICS	-1.8	ARHGAP1	-1.6	SGEF	-1.8
SPATA13	-1.7	ARHGEF5	-1.6	DOCK1	-1.6
DOCK4	-1.6	FLJ43692	-1.6	FLJ13815	-1.6
RACGAP1	-1.6	C9ORF100	-1.5	ARHGAP22	-1.5
OCRL	-1.6	SGEF	-1.5	PIK3R2	1.5
ARHGEF4	-1.6	FGD1	-1.5	ALS2	1.5
CENTD3	-1.6	VAV2	1.6	ITSN2	1.6
TIAM2	1.5	CHN2	1.6	ARHGEF9	1.8
ARHGAP24	1.6	ARHGAP10	1.9	FAM13A1	1.9
DOCK1	1.7	ARHGAP22	1.9	ABR	1.9
FGD1	1.8	OPHN1	2.0	ECT2	1.9
ARHGEF35	1.9	AKAP13	2.0	RICS	1.9
ARHGAP20	1.9	OCRL	2.0	TRIO	2.6
SGEF	2.0	SPATA13	2.0	CENTD3	2.8
ARHGAP1	2.0	ARHGEF4	2.3		
FLJ13815	2.1	GEFT	2.4		

Table S4 related to figure 2: Candidate genes from siGENOME screen on top of soft collagen.

Gene	Z Score Round	Gene	Z Score Spindle	Gene	Z score Star
ECT2	-2.2	ARHGAP32	-1.6	STARD13	-3.0
ARHGEF9	-2.0	ARHGAP18	1.7	VAV3	-2.5
ITSN2	-1.6	DOCK5	2.1	SRGAP1	-2.3
PREX2	-1.5	ECT2	2.1	ARHGEF38	-2.0
ARHGEF7	1.6	ITSN2	2.2	ARHGAP36	-1.9
ARHGEF35	1.6	CHN2	2.4	FARP1	-1.8
TIAM2	1.6	PREX2	2.9	ARAP3	-1.8
FGD3	1.7	FAM13A	3.1	ARHGAP32	-1.8
ARHGAP36	1.8	ARHGEF9	3.7	TIAM2	-1.8
TRIO	1.8			PLEKHG2	-1.7
ARHGEF38	1.9			TRIO	-1.7
ARHGAP32	1.9			ARHGEF35	-1.6
FARP1	1.9			FGD3	-1.6
PLEKHG2	2.1			AKAP13	1.5
VAV3	2.1			FAM13B	1.6
SRGAP1	2.5			VAV1	1.6
STARD13	2.8			RACGAP1	1.8
				ECT2	1.8

Table S5 related to figure 2: Candidate genes from OTP screen on top of soft collagen.

Final population - Number of Objects
Final population - Intensity Nucleus Exp1Cam1 Mean - Mean per Well
Final population - Intensity Cell Exp2Cam2 Mean - Mean per Well
Final population - Nucleus Area [ÂµmÂ²] - Mean per Well
Final population - Nucleus Roundness - Mean per Well
Final population - Nucleus Width [Âµm] - Mean per Well
Final population - Nucleus Length [Âµm] - Mean per Well
Final population - Nucleus Ratio Width to Length - Mean per Well
Final population - Nucleus STAR morphology Symmetry 02 - Mean per Well
Final population - Nucleus STAR morphology Symmetry 03 - Mean per Well
Final population - Nucleus STAR morphology Symmetry 04 - Mean per Well
Final population - Nucleus STAR morphology Symmetry 05 - Mean per Well
Final population - Nucleus STAR morphology Symmetry 12 - Mean per Well
Final population - Nucleus STAR morphology Symmetry 13 - Mean per Well
Final population - Nucleus STAR morphology Symmetry 14 - Mean per Well
Final population - Nucleus STAR morphology Symmetry 15 - Mean per Well
Final population - Nucleus STAR morphology Threshold Compactness 30% - Mean per Well
Final population - Nucleus STAR morphology Threshold Compactness 30% - StdDev per Well
Final population - Nucleus STAR morphology Threshold Compactness 40% - Mean per Well
Final population - Nucleus STAR morphology Threshold Compactness 40% - StdDev per Well
Final population - Nucleus STAR morphology Threshold Compactness 50% - Mean per Well
Final population - Nucleus STAR morphology Threshold Compactness 50% - StdDev per Well
Final population - Nucleus STAR morphology Threshold Compactness 60% - Mean per Well
Final population - Nucleus STAR morphology Threshold Compactness 60% - StdDev per Well
Final population - Nucleus STAR morphology Axial Small Length - Mean per Well
Final population - Nucleus STAR morphology Axial Small Length - StdDev per Well
Final population - Nucleus STAR morphology Axial Length Ratio - Mean per Well
Final population - Nucleus STAR morphology Axial Length Ratio - StdDev per Well
Final population - Nucleus STAR morphology Radial Mean - Mean per Well
Final population - Nucleus STAR morphology Radial Mean - StdDev per Well
Final population - Nucleus STAR morphology Radial Relative Deviation - Mean per Well
Final population - Nucleus STAR morphology Radial Relative Deviation - StdDev per Well
Final population - Nucleus STAR morphology Profile 1/5 - Mean per Well
Final population - Nucleus STAR morphology Profile 1/5 - StdDev per Well
Final population - Nucleus STAR morphology Profile 2/5 - Mean per Well
Final population - Nucleus STAR morphology Profile 2/5 - StdDev per Well
Final population - Nucleus STAR morphology Profile 3/5 - Mean per Well
Final population - Nucleus STAR morphology Profile 3/5 - StdDev per Well
Final population - Nucleus STAR morphology Profile 4/5 - Mean per Well
Final population - Nucleus STAR morphology Profile 4/5 - StdDev per Well
Final population - Nucleus STAR morphology Profile 5/5 - Mean per Well
Final population - Nucleus STAR morphology Profile 5/5 - StdDev per Well
Final population - Cell STAR morphology Symmetry 02 - Mean per Well
Final population - Cell STAR morphology Symmetry 02 - StdDev per Well
Final population - Cell STAR morphology Symmetry 03 - Mean per Well
Final population - Cell STAR morphology Symmetry 03 - StdDev per Well
Final population - Cell STAR morphology Symmetry 04 - Mean per Well
Final population - Cell STAR morphology Symmetry 04 - StdDev per Well
Final population - Cell STAR morphology Symmetry 05 - Mean per Well
Final population - Cell STAR morphology Symmetry 05 - StdDev per Well
Final population - Cell STAR morphology Symmetry 12 - Mean per Well
Final population - Cell STAR morphology Symmetry 12 - StdDev per Well
Final population - Cell STAR morphology Symmetry 13 - Mean per Well
Final population - Cell STAR morphology Symmetry 13 - StdDev per Well
Final population - Cell STAR morphology Symmetry 14 - Mean per Well
Final population - Cell STAR morphology Symmetry 14 - StdDev per Well
Final population - Cell STAR morphology Symmetry 15 - Mean per Well
Final population - Cell STAR morphology Symmetry 15 - StdDev per Well

Final population - Cell STAR morphology Threshold Compactness 30% - Mean per Well
Final population - Cell STAR morphology Threshold Compactness 30% - StdDev per Well
Final population - Cell STAR morphology Threshold Compactness 40% - Mean per Well
Final population - Cell STAR morphology Threshold Compactness 40% - StdDev per Well
Final population - Cell STAR morphology Threshold Compactness 50% - Mean per Well
Final population - Cell STAR morphology Threshold Compactness 50% - StdDev per Well
Final population - Cell STAR morphology Threshold Compactness 60% - Mean per Well
Final population - Cell STAR morphology Threshold Compactness 60% - StdDev per Well
Final population - Cell STAR morphology Axial Small Length - Mean per Well
Final population - Cell STAR morphology Axial Small Length - StdDev per Well
Final population - Cell STAR morphology Axial Length Ratio - Mean per Well
Final population - Cell STAR morphology Axial Length Ratio - StdDev per Well
Final population - Cell STAR morphology Radial Mean - Mean per Well
Final population - Cell STAR morphology Radial Mean - StdDev per Well
Final population - Cell STAR morphology Radial Relative Deviation - Mean per Well
Final population - Cell STAR morphology Radial Relative Deviation - StdDev per Well
Final population - Cell STAR morphology Profile 1/5 - Mean per Well
Final population - Cell STAR morphology Profile 1/5 - StdDev per Well
Final population - Cell STAR morphology Profile 2/5 - Mean per Well
Final population - Cell STAR morphology Profile 3/5 - Mean per Well
Final population - Cell STAR morphology Profile 3/5 - StdDev per Well
Final population - Cell STAR morphology Profile 4/5 - Mean per Well
Final population - Cell STAR morphology Profile 4/5 - StdDev per Well
Final population - Cell STAR morphology Profile 5/5 - Mean per Well
Final population - Cell STAR morphology Profile 5/5 - StdDev per Well
Final population - Nucleus Exp1Cam1 SER Spot 0 px - Mean per Well
Final population - Nucleus Exp1Cam1 SER Spot 0 px - StdDev per Well
Final population - Nucleus Exp1Cam1 SER Hole 0 px - Mean per Well
Final population - Nucleus Exp1Cam1 SER Hole 0 px - StdDev per Well
Final population - Nucleus Exp1Cam1 SER Edge 0 px - Mean per Well
Final population - Nucleus Exp1Cam1 SER Edge 0 px - StdDev per Well
Final population - Nucleus Exp1Cam1 SER Ridge 0 px - Mean per Well
Final population - Nucleus Exp1Cam1 SER Ridge 0 px - StdDev per Well
Final population - Nucleus Exp1Cam1 SER Valley 0 px - Mean per Well
Final population - Nucleus Exp1Cam1 SER Valley 0 px - StdDev per Well
Final population - Nucleus Exp1Cam1 SER Saddle 0 px - Mean per Well
Final population - Nucleus Exp1Cam1 SER Saddle 0 px - StdDev per Well
Final population - Nucleus Exp1Cam1 SER Bright 0 px - Mean per Well
Final population - Nucleus Exp1Cam1 SER Bright 0 px - StdDev per Well
Final population - Nucleus Exp1Cam1 SER Dark 0 px - Mean per Well
Final population - Nucleus Exp1Cam1 SER Dark 0 px - StdDev per Well
Final population - Nucleus Exp1Cam1 Haralick Correlation 1 px - Mean per Well
Final population - Nucleus Exp1Cam1 Haralick Correlation 1 px - StdDev per Well
Final population - Nucleus Exp1Cam1 Haralick Contrast 1 px - Mean per Well
Final population - Nucleus Exp1Cam1 Haralick Contrast 1 px - StdDev per Well
Final population - Nucleus Exp1Cam1 Haralick Sum Variance 1 px - Mean per Well
Final population - Nucleus Exp1Cam1 Haralick Sum Variance 1 px - StdDev per Well
Final population - Nucleus Exp1Cam1 Haralick Homogeneity 1 px - Mean per Well
Final population - Nucleus Exp1Cam1 Haralick Homogeneity 1 px - StdDev per Well
Final population - Nucleus Exp1Cam1 Gabor Min 2 px w2 - Mean per Well
Final population - Nucleus Exp1Cam1 Gabor Min 2 px w2 - StdDev per Well
Final population - Nucleus Exp1Cam1 Gabor Max 2 px w2 - Mean per Well
Final population - Nucleus Exp1Cam1 Gabor Max 2 px w2 - StdDev per Well
Final population - Cell Exp2Cam2 SER Spot 0 px - Mean per Well
Final population - Cell Exp2Cam2 SER Spot 0 px - StdDev per Well
Final population - Cell Exp2Cam2 SER Hole 0 px - Mean per Well
Final population - Cell Exp2Cam2 SER Hole 0 px - StdDev per Well
Final population - Cell Exp2Cam2 SER Edge 0 px - Mean per Well

Final population - Cell Exp2Cam2 SER Edge 0 px - StdDev per Well
Final population - Cell Exp2Cam2 SER Ridge 0 px - Mean per Well
Final population - Cell Exp2Cam2 SER Ridge 0 px - StdDev per Well
Final population - Cell Exp2Cam2 SER Valley 0 px - Mean per Well
Final population - Cell Exp2Cam2 SER Valley 0 px - StdDev per Well
Final population - Cell Exp2Cam2 SER Saddle 0 px - Mean per Well
Final population - Cell Exp2Cam2 SER Saddle 0 px - StdDev per Well
Final population - Cell Exp2Cam2 SER Bright 0 px - Mean per Well
Final population - Cell Exp2Cam2 SER Bright 0 px - StdDev per Well
Final population - Cell Exp2Cam2 SER Dark 0 px - Mean per Well
Final population - Cell Exp2Cam2 SER Dark 0 px - StdDev per Well
Final population - Cell Exp2Cam2 Haralick Correlation 1 px - Mean per Well
Final population - Cell Exp2Cam2 Haralick Correlation 1 px - StdDev per Well
Final population - Cell Exp2Cam2 Haralick Contrast 1 px - Mean per Well
Final population - Cell Exp2Cam2 Haralick Contrast 1 px - StdDev per Well
Final population - Cell Exp2Cam2 Haralick Sum Variance 1 px - Mean per Well
Final population - Cell Exp2Cam2 Haralick Sum Variance 1 px - StdDev per Well
Final population - Cell Exp2Cam2 Haralick Homogeneity 1 px - Mean per Well
Final population - Cell Exp2Cam2 Haralick Homogeneity 1 px - StdDev per Well
Final population - Cell Exp2Cam2 Gabor Min 2 px w2 - Mean per Well
Final population - Cell Exp2Cam2 Gabor Min 2 px w2 - StdDev per Well
Final population - Cell Exp2Cam2 Gabor Max 2 px w2 - Mean per Well
Final population - Cell Exp2Cam2 Gabor Max 2 px w2 - StdDev per Well
Final population - Number of Objects
Final population - Intensity Nucleus Exp1Cam1 Mean - Mean per Well
Final population - Intensity Cell Exp2Cam2 Mean - Mean per Well
Final population - Nucleus Area [ÂµmÂ²] - Mean per Well
Final population - Nucleus Roundness - Mean per Well
Final population - Nucleus Width [Âµm] - Mean per Well

Table S6 related to figure 3: Morphological and texture features, Screens on top of plastic.

Gene	Z score Spindle	Gene	Z score Small Flat	Gene	Z score Big Flat
MYO9B	-2.3	C5ORF5	-2.0	STARD13	1.5
DOCK9	-2.2	GMIP	-1.7	ARHGAP23	1.6
ITSN2	-2.0	7H3	-1.7	MYO9A	1.6
CDGAP	-2.0	DOCK9	1.5	DOCK11	1.6
ARHGEF9	-1.9	ARHGAP6	1.7	ARHGAP12	1.7
DOCK11	-1.9	CDGAP	1.9	BCR	1.7
ARHGAP8	-1.8	LOC343578	2.2	7H3	1.8
LOC343578	-1.7	MYO9B	4.3	ABR	2.3
LOC345930	-1.7			ARHGAP5	2.3
ARHGAP23	-1.6			ARHGAP8	3.3
ARHGAP6	-1.5			ARHGEF9	5.3
GMIP	1.5				
ARHGEF18	1.5				
SGEF	1.7				
SOS2	1.7				
PLEKHG4	1.8				
DEF6	1.9				
NET1	1.9				
ARHGEF15	2.0				
PIK3R2	2.2				
ARHGAP26	2.3				
ARHEGF35	2.6				

Table S7 related to figure 3: Candidate genes from siGENOME screen on top of plastic.

Gene	Z score Spindle	Gene	Z score Small Flat	Gene	Z score Big Flat
ECT2	-2.6	DOCK8	-2.3	FARP1	-3.0
STARD13	-1.6	ARHGEF5	-2.2	ARHGEF38	-1.8
HMHA1	-1.6	ARHGAP18	-2.2	PLEKHG2	-1.7
ARHGEF1	-1.6	FARP1	-1.8	ARHGAP15	-1.6
TRIO	-1.5	ARHGEF9	-1.8	ARHGEF18	-1.6
SOS1	1.5	ARHGEF2	-1.6	ARHGEF9	1.7
ARHGEF10	1.6	ARHGEF39	-1.5	AKAP13	1.8
ARHGAP35	1.6	ARHGEF1	1.7	CHN2	1.9
ARFGEF3	1.6	ARHGAP33	1.7	ITSN2	2.1
ARHGEF2	1.8	TIAM2	2.1	ARHGEF40	2.3
MCF2	1.8	STARD13	2.7		
ARHGEF35	1.8				
ARHGAP18	2.5				

Table S8 related to figure 3: Candidate genes from OTP screen on top of plastic.
